# Microarray analyses reveal novel targets of exercise-induced stress resistance in the dorsal raphe nucleus

**DOI:** 10.3389/fnbeh.2013.00037

**Published:** 2013-05-10

**Authors:** Alice B. Loughridge, Benjamin N. Greenwood, Heidi E. W. Day, Matthew B. McQueen, Monika Fleshner

**Affiliations:** ^1^Department of Integrative Physiology, University of Colorado BoulderBoulder, CO, USA; ^2^The Center for Neuroscience, University of Colorado BoulderBoulder, CO, USA; ^3^Department of Psychology and Neuroscience, University of Colorado BoulderBoulder, CO, USA; ^4^Institute for Behavioral Genetics, University of Colorado BoulderBoulder, CO, USA

**Keywords:** Affymetrix gene microarray, Weighted Gene Correlational Network Analysis, bioinformatics, laser capture microdissection, stress resistance, dorsal raphe nucleus

## Abstract

Serotonin (5-HT) is implicated in the development of stress-related mood disorders in humans. Physical activity reduces the risk of developing stress-related mood disorders, such as depression and anxiety. In rats, 6 weeks of wheel running protects against stress-induced behaviors thought to resemble symptoms of human anxiety and depression. The mechanisms by which exercise confers protection against stress-induced behaviors, however, remain unknown. One way by which exercise could generate stress resistance is by producing plastic changes in gene expression in the dorsal raphe nucleus (DRN). The DRN has a high concentration of 5-HT neurons and is implicated in stress-related mood disorders. The goal of the current experiment was to identify changes in the expression of genes that could be novel targets of exercise-induced stress resistance in the DRN. Adult, male F344 rats were allowed voluntary access to running wheels for 6 weeks; exposed to inescapable stress or no stress; and sacrificed immediately and 2 h after stressor termination. Laser capture micro dissection selectively sampled the DRN. mRNA expression was measured using the whole genome Affymetrix microarray. Comprehensive data analyses of gene expression included differential gene expression, log fold change (LFC) contrast analyses with False Discovery Rate correction, KEGG and Wiki Web Gestalt pathway enrichment analyses, and Weighted Gene Correlational Network Analysis (WGCNA). Our results suggest that physically active rats exposed to stress modulate expression of twice the number of genes, and display a more rapid and strongly coordinated response, than sedentary rats. Bioinformatics analyses revealed several potential targets of stress resistance including genes that are related to immune processes, tryptophan metabolism, and circadian/diurnal rhythms.

## Introduction

Depression and anxiety frequently coexist and are the most common mood disorders affecting society. The World Health Organization estimates that 121 million people currently suffer from depression. Individuals suffering from depression have significant impairment in quality of life (Rapaport et al., 2005), are at increased risk for developing coronary heart disease (Wulsin and Singal, 2003) and type 2 diabetes (Knol et al., 2006), and have higher mortality due to suicide. By 2030, depression is expected to be a leading cause in the global burden of disease (Mathers and Loncar, 2006).

Stressful life events often precede the onset of depression (Kendler et al., 1999; van Praag, 2005) and anxiety. Despite the high occurrence and significant disability associated with stress-related mood disorders, the pathophysiology of these conditions is not fully understood. Important to note is that not every individual who experiences a stressful life event develops a serious mood disorder, and these individuals may possess resistance to the negative affective consequences of stress. Pinpointing the factors by which stress resistance occurs could provide a better understanding of the neurobiological mechanisms underlying stress-related mood disruptions.

To investigate the neural circuitry underlying stress-related mood disorders, researchers use animal models (Krishnan and Nestler, 2008). Rats exposed to an acute inescapable stressor, such as tail shock, later exhibit behaviors argued to resemble symptoms of human anxiety and depression (Maier and Watkins, 1998), and these behaviors are responsive to pharmaceutical treatment with anxiolytics (Drugan et al., 1984) and antidepressants (Sherman et al., 1982). Inescapable stressor exposure also produces various physiological perturbations. Long-term increases in basal levels of plasma corticosterone and decreases in corticosteroid-binding globulin occur in rats following tail shock (Fleshner et al., 1995). Additionally, acute stress increases interleukin-1β (IL-1β), leading to immune modulation (Moraska et al., 2002), and centrally, contributes to behavioral consequences of stress (Maier and Watkins, 1995). Circadian-regulated processes are also susceptible to acute stress. Thompson et al. (2013) observed a decrease in amplitude and disruption in diurnal pattern of core body temperature and heart rate in rats exposed to tail shock. Moreover, inescapable stress produces alterations in brain serotonergic circuits. The serotonergic system has long been implicated in underlying the behavioral consequences of inescapable stress exposure in rats (Maier and Watkins, 2005) and has been heavily implicated in human affective disorders (Sharp and Cowen, 2011).

Numerous components of the serotonergic system such as serotonin (5-HT) receptors, the 5-HT transporter, and extracellular 5-HT levels are sensitive to stress. Serotonergic nerve terminals and receptors also occupy regions of the brain involved in neuroendocrine and behavioral responses to stress (Chaouloff, 1993). One region of particular interest is the dorsal raphe nucleus (DRN), a small midbrain structure containing a high concentration of stress-responsive 5-HT cell bodies (Grahn et al., 1999). Hyper activation and sensitization of DRN 5-HT neurons is thought to underlie the depression- and anxiety-like behaviors induced by inescapable stress exposure (Maier et al., 1995; Christianson et al., 2008).

The DRN receives afferent, and provides efferent, projections to brain regions involved in fear, anxiety, and depression. These regions include the prefrontal cortex, striatum, bed nucleus of the stria terminalis (BNST), amygdala, and locus coeruleus (LC). Efferent DRN projections render these regions susceptible to stress-induced 5-HT activity in the DRN. Furthermore, these regions are themselves sensitive to stress (Cullinan et al., 1995), provide afferent input to the DRN, and may modulate DRN 5-HT activity. Nerve terminals containing corticotropin-releasing factor (CRF), a neuropeptide produced in response to elevated cortisol levels, for example, are present in the DRN (Swanson et al., 1983). Given that the BNST projects to the DRN and contains many CRF neurons (Day et al., 1999), the BNST is believed to be a primary source of CRF to the DRN. Interestingly, CRF injected into the DRN increases 5-HT activity in a subpopulation of cells (Lowry et al., 2000), and injection of CRF into the caudal DRN produces behaviors resembling those produced by inescapable stress exposure (Hammack et al., 2002). Thus other brain regions influence DRN 5-HT levels, and interactions between those regions and the DRN likely contribute to the DRN's role in stress-related mood disorders.

Also important to consider is that within the DRN, interactions between diverse cell populations may influence stress-induced 5-HT activity. The DRN is not just a homogenous structure of 5-HT neurons. Other populations of neurons containing the neurotransmitters γ-aminobutyric acid (Belin et al., 1979; Day et al., 2004), dopamine (Lindvall and Björklund, 1974; Stratford and Wirtshafter, 1990), and glutamate (Commons et al., 2005) also exist. Cells containing neuropeptides such as substance P (Hökfelt et al., 1978) and neuropeptide Y (de Quidt and Emson, 1986) are also present. These various neuropeptides and neurotransmitters/receptors are capable of modulating 5-HT (Ferré et al., 1994; Song et al., 1996; Tao and Auerbach, 2000; Valentino et al., 2003). Therefore, stress-induced alterations in 5-HT activity within the DRN and at DRN projection sites may be influenced indirectly through non-serotonergic neuronal modulation of serotonergic neurons. Non-serotonergic neurons in the DRN are also sensitive to 5-HT, and can have inhibitory and excitatory responses to 5-HT release (Marinelli et al., 2004). Dynamic interactions between serotonergic and non-serotonergic neurons originating at DRN afferent sites and within the DRN likely contribute to the effect of stress on net DRN 5-HT release within the DRN and at DRN projections sites.

Non-neuronal cell types, such as astrocytes and microglia, may also influence DRN neural activity. Microglia are the resident “immune cells” of the brain and are sensitive to stress-induced elevation of glucocorticoids (Nair and Bonneau, 2006; Sugama et al., 2007). Activated microglia release interleukin-1 (IL-1) (Giulian et al., 1986), tumor necrosis factor-α (TNF-α) (Sawada et al., 1989), and interleukin-6 (IL-6) (Righi et al., 1989). Inescapable stress increases IL-1β in the brain (Nguyen et al., 1998). Stress-induced activation of microglia may occur in the DRN and effect 5-HT neurons. Consistent with this idea, administration of interferon-γ (IFN-γ) and TNF-α reduced the survival of 5-HT neurons in organotypic DRN sections (Hochstrasser et al., 2011).

Overall, the DRN is an important region of investigation in studying the neurobiological mechanisms of stress-related mood disorders. Elucidation of variables influencing the serotonergic response to stress within the DRN may provide a better understanding of the development of these disorders. Furthermore, identification of interventions that prevent or manipulate the serotonergic response to stress and/or influence the various factors capable of modulating 5-HT activity within the DRN, may lead to the identification of novel therapeutic targets.

In humans, physical activity is one factor known to influence an individual's response to stress. Exercise reduces the risk of developing stress-related depression and anxiety (Fox, 1999). Similarly, in rats, 6 weeks of voluntary wheel running protects against the behavioral consequences of inescapable stress exposure (Greenwood et al., 2003). It is believed that wheel running prevents these behaviors by attenuating stress-induced activation of 5-HT neurons within the DRN. Wheel running may do this by producing plasticity at (1) DRN afferent sites (2) DRN efferent sites or (3) within the DRN itself (Greenwood and Fleshner, 2011). Given that hyperactivity of 5-HT neurons in the DRN is necessary for the development of stress-induced behaviors in rats and our lab has previously shown that wheel running attenuates stress-induced c-fos expression in DRN 5-HT neurons (Greenwood et al., 2003), we will focus on exercise-induced plastic changes that may occur within the DRN itself.

In particular, the 5-HT_1*A*_ inhibitory autoreceptor has been implicated in the mechanism by which wheel running could constrain stress-induced 5-HT activity and protect against the behavioral consequences of inescapable stress. 5-HT_1*A*_ receptors inhibit the activity of 5-HT neurons (Sprouse and Aghajanian, 1987) and reduce 5-HT release (Casanovas et al., 1997). Six weeks of wheel running increases 5-HT_1*A*_ mRNA expression in the DRN (Greenwood et al., 2003, 2005) and thus, may increase 5-HT_1*A*_ receptor-mediated inhibition of DRN 5-HT neurons during inescapable stress.

The protective effect of wheel running could also occur indirectly, through a non-serotonergic route. One possibility is through neuropeptides. Wheel running increases brain-derived neurotropic factor (BDNF) (Neeper et al., 1995), a neuropeptide important for maintaining neuronal health and function, and galanin (Tong et al., 2001) in the hippocampus, and also upregulates gene expression of galanin in the LC (Holmes et al., 2006; Sciolino et al., 2012). Wheel running may also increase levels of BDNF and galanin in the DRN. Both factors are coexpressed in 5-HT neurons in the DRN (Merlio et al., 1992; Xu and Hökfelt, 1997) and are capable of modulating 5-HT activity. Infusion of BDNF into the DRN modifies the neuronal firing of 5-HT by decreasing the regularity of the firing pattern (Celada et al., 1996). Additionally, an *in vitro* study revealed that galanin hyperpolarizes 5-HT neurons within the DRN (Xu et al., 1998). Exercise-induced increases in BDNF and galanin may protect against stress-induced activation of 5-HT neurons through modulating and, in the case of BDNF, inhibiting 5-HT neuronal activity.

Another method by which exercise may confer protection is through an immune-related mechanism. Evidence suggests a role of cytokines in human mood disorders (Maes, 2008) and stress-induced behaviors in rats. Injection of an IL-1 receptor antagonist into the brain blocks stress-induced depression- and anxiety-like behaviors in rats (Maier and Watkins, 1995), suggesting that activity at brain IL-1 receptors is important for the production of these behaviors. Speaker et al. (2011) observed that 6 weeks of wheel running attenuates stress-induced increases in plasma IL-1β, one of two cytokines that bind IL-1 receptors. It is possible that 6 weeks of wheel running also reduces stress-induced increases in brain IL-1β, and through reducing ligand availability, protects against stress-induced alterations in brain IL-1 receptor activity. Given that the DRN contains many IL-1 receptors (Cunningham and De Souza, 1993), it may be particularly sensitive to stress-induced and/or exercise-induced alterations in IL-1 receptor activity.

Though the precise mechanisms are not fully understood, the protective effect of exercise likely involves preventing stress-induced alterations in the serotonergic system, either by directly constraining activity of 5-HT neurons within the DRN or indirectly, through altering other neurotransmitter systems or neuropeptides within the DRN that are capable of modulating 5-HT neurons. Furthermore, DRN 5-HT neurons may be influenced by exercise-induced plastic changes that reduce afferent input to the DRN, activate afferent inhibition of the DRN during stress (Greenwood and Fleshner, 2011), or produce alterations in postsynaptic 5-HT receptor function (Greenwood et al., 2012). Elucidating the mechanism by which exercise produces stress-resistance and protects against the behavioral consequences of stress may lead to the identification of novel therapeutic targets and development of more targeted drugs for the treatment of human stress-related mood disorders.

One approach to reveal novel targets is by employing the use of microarray technology. Microarray technology permits the investigation of the expression of tens of thousands of genes simultaneously, at the level of mRNA transcription. Predesigned chips that contain sequences, known as probes, derived from every gene within a specified genome can be probed with mRNAs obtained from experimental samples in order to gain information about gene expression under the given conditions (Cox et al., 2012). When used in conjunction with laser capture microdissection, microarrays can reveal expression patterns of genes within specific cells. Using microarray and laser capture microdissection, therefore, it is possible to assess the effect of exercise and/or stress on gene expression in cell populations specific to the DRN.

The purpose of this experiment was to investigate the effect of exercise and/or stress on gene expression within the DRN. We hypothesized that wheel running produces changes in mRNA transcription within the DRN, and physically active rats exposed to stress have different gene expression profiles compared to sedentary rats exposed to stress. The differences in gene expression patterns within the DRN between physically active and sedentary rats exposed to stress may underlie the molecular mechanisms by which exercise protects against behaviors produced by inescapable stress exposure. Whole genome Affymetrix microarray analysis was used to assess gene expression. Our goal was to use an exploratory approach to (1) systematically organize the transcriptome (17,170 genes) obtained from the microarray analysis into a more manageable and focused gene set and (2) extrapolate physiological implications from this focused gene set by identifying novel targets of exercise-induced stress resistance within the DRN. To ensure a comprehensive assessment of the data, the organizational process involved two approaches, (1) identification of genes based on changes in differential expression in response to exercise and/or stress (2) identification of genes based on changes in coexpression in response to exercise and/or stress. For the differential expression analysis, two measures of significance were utilized. In a more conservative approach, genes were identified by log fold changes in gene expression. In a second, less stringent approach, genes statistically significantly differentially expressed by *p* < 0.05 were identified. These *p*-values were corrected for multiple comparisons using the False Discovery Rate adjustment method. The coexpression analysis narrowed the transcriptome from 17,170 genes to 11 modules of highly coexpressed networks of genes. These networks of genes were then correlated to the response to exercise and/or stress. Both the differential and coexpression analysis returned sets of genes that were further sorted by their relationship to functional categories derived from bioinformatics databases. Novel targets of exercise-induced stress resistance were identified within these functional categories.

## Materials and methods

### Animals

The University of Colorado Boulder Animal Care and Use Committee approved all protocols for this study. A total of 48 adult, male Fisher 344 rats weighing 170–180 grams at time of arrival (Harlan Laboratories) were used in this experiment. Upon arrival, animals were individually housed in Nalgene Plexiglas cages (45 × 25.2 × 14.7 cm). The housing environment was maintained on a 12:12 h light:dark cycle, controlled for humidity, and held at a constant temperature of 22°C. Rats were allowed ad libitum access to food and water and were weighed weekly to ensure each animal remained healthy. Following arrival, animals were acclimated to the housing conditions for 1 week before experimental manipulation.

### Wheel running

Animals were randomly assigned to remain sedentary (Sed, *n* = 23) or were housed with a running wheel (Run, *n* = 25), and allowed voluntary access to the wheel for 6 weeks. During the 1-week acclimation period, wheels were rendered immobile with metal stakes. Daily wheel revolutions each animal ran were logged with Vital View software (Mini Mitter). The product of the total number of daily revolutions and the wheel circumference (1.081 m) was calculated to obtain daily running distance. Daily running distance was summed in order to get an average weekly running distance.

### Inescapable stress

Animals were randomly assigned to remain in their home cages (HC) or receive inescapable tail shock (Stress). The stress procedure occurred between 0700 and 1200. Animals subjected to stress were restrained in acrylic cylinders (23.4 × 7 cm diameter). The tail projected from the back of the restraint device. An electrode was positioned 3 cm from the base of the tail and served as the vehicle by which shock was delivered. The shock procedure consisted of 100, 5-s tail shocks administered on a random 60-s inter-trial interval. Rats received 1.0 mA tail shocks for 50 min, at which time the intensity of shock was increased to 1.5 mA tail shocks for the remainder of the session. The entire stress procedure lasted 1 h and 48 min. Rats were sacrificed by rapid decapitation immediately following termination of tail shock (Stress0) or 2 h post termination of tail shock (Stress2). The sacrificing of rats that remained in their home cage was time matched with those animals subjected to tail shock.

### Tissue collection and cryosectioning

RNAse free conditions were maintained throughout tissue processing. After rats were sacrificed, brains were extracted and flash frozen at −20°C, in 2-Methylbutane (Fisher Scientific), for 4 min. Brains were stored at −80°C prior to sectioning. Brains were prepared with M-1 embedding matrix before sectioning at −21°C with a cryostat (Leica CM1850). Tissue was sectioned to a thickness of 20 um through the rostral to mid-caudal (approximately −7.3 to −8.2 mm relative to Bregma) portions of the DRN. This specific region of the DRN was targeted because it is involved in modulating stress- and anxiety-like behaviors (Hale et al., 2012) and prior evidence suggests that alterations in gene expression occur in this region following 6 weeks of wheel running (Greenwood et al., 2003, 2005). Sections were freeze-mounted to PEN membrane frame slides (MDS Analytical Technologies) and stored at −80°C until further use.

### Laser capture microdissection and RNA isolation

Laser capture microdissection was performed to procure a precise sample of the DRN from each rat. Slides containing sections of DRN were allowed to thaw for 20 s prior to being fixed in 75% ethanol, subjected to a Histogene Stain (for visualization purposes), and dehydrated in graded ethanol concentrations, in accordance with the Arcturus Histogene LCM Frozen Section Staining Kit protocols (Applied Biosystems). Following staining procedures, slides were loaded into the laser capture microdissection system (Arcturus XT, Life Technologies). The regions of DRN targeted for capture were the dorsal and ventral portions of the rostral to mid-caudal DRN. Samples were captured so that each sample contained the entire portion of the dorsal and ventral portion of the DRN at the given rostral-caudal level. DRN samples were obtained by using an infrared laser to adhere the tissue to a cap coated with a thermoplastic film (Capsure Macro LCM Caps, Applied Biosystems). An ultraviolet laser was used to separate the DRN from the rest of the brain section. An average of 23 DRN samples, ranging in size from 300,000 to 800,000 um^2^ (depending on rostral to caudal level), were successfully dissected and pooled for each rat to ensure maximal total RNA yield. Following laser capture microdissection, caps were incubated in RNA extraction buffer (Applied Biosystems) for 30 min and frozen at −80°C until future use. RNA was isolated using the Arcturus Picopure RNA Isolation Kit (Applied Biosystems) in accordance with kit protocols. Samples were stored in Elution Buffer (Applied Biosystems) at −80°C until microarray analysis.

### Microarray analysis

Samples were sent to the Genomics and Microarray Core Facility at the University of Colorado Denver for whole genome analysis using microarray. RNA integrity was evaluated with the Agilent Bioanalyzer 2100 and RNA 6000 Nano/Pico Kit (Agilent Technologies). Concentrations of extracted RNA were assessed with the Nanodrop spectrophotometer (Nanodrop Technologies). One sample was removed from further processing due to poor integrity of RNA (*n* = 47). A total of 100–150 ug RNA per each sample was converted to double stranded cDNA and then transcribed into cRNA using the Ambion WT Expression Kit, in accordance with kit protocols. Following generation of cRNA, second cycle, first strand cDNA synthesis was carried out in order to transform the cRNA into single-strand cDNA. The cDNA was fragmented and the Genechip WT Terminal Labeling Kit (Affymetrix) was used to label the single-stranded DNA with biotin. Samples were hybridized to an Affymetrix Genechip Rat Gene 1.1 ST Array Platform. Hybridization, washing, staining, and scanning were executed using the GeneTitan instrument (Affymetrix).

### Microarray data pre-processing

The Bioconductor toolset within the R statistical software program was used to format the raw microarray data. This pre-processing was completed using the ‘expresso’ option in the ‘affy’ package of the Bioconductor toolset and included background adjustment, log fold transformation, and normalization. To control for inter-array variability, the dataset was normalized using the Robust Multi Array Average method. Gene chip and RNA quality were assessed by examining total mRNA expression for each animal.

### Microarray contrast generation

Following pre-processing and normalization, a standardized expression value was obtained for each gene for each rat. The expression values for each gene were averaged for each experimental group. The LIMMA package was used to generate nine contrasts between experimental groups. These contrasts included [(Sed_Stress0_ v. Sed_HC_) v. (Run_Stress0_ v. Run_HC_)], [(Sed_Stress2_ v. Sed_HC_) v. (Run_Stress2_ v. Run_HC_)], Run_HC_ v. Sed_HC_, Sed_Stress0_ v. Sed_HC_, Sed_Stress2_ v. Sed_HC_, Run_Stress0_ v. Run_HC_, Run_Stress2_ v. Run_HC_, Run_Stress0_ v. Sed_Sress0_, and Run_Stress2_ v. Sed_Sress2_. For each contrast, the difference in the expression level of each individual gene was calculated by subtracting the expression level of the 2^nd^ group in each contrast from the expression level of the 1^st^ group in each contrast. For example, the contrast Run_HC_ v. Sed_HC_ indicates that the expression level of gene X in the Sed_HC_ group was subtracted from the expression level of gene X in the Run_HC_ group, or (Run_HC_—Sed_HC_). The first two contrasts represent the interaction between exercise and stress at each time point. For each contrast, *p*-values and test statistics were calculated for each gene according to the absolute value of difference in gene expression observed between the groups. The False Discovery Rate multiple-test adjustment method was applied in the calculation of these *p*-values in order to control for the chance of yielding false positive (significant) results. The log fold change (LFC) in gene expression was also calculated for each gene in each contrast. Out of 27,000 possible genes, 17,170 gene transcripts were reliably detected. These genes were considered the transcriptome, or the genes expressed in cells of the DRN as a result of the experimental conditions.

### Differential gene expression identification and bioinformatics

In an initial approach, genes differentially expressed by a LFC ≥ ± 1.1 were identified for each contrast. A second approach was performed utilizing the same contrasts as previously described. However, less stringent requirements for statistical significance were utilized (*p* < 0.05) to identify differentially expressed genes between groups. Genes that were significantly differentially expressed at a *p* < 0.05 were organized into nine sets, one set for each contrast, and imported into the bioinformatics system, Web Gestaldt. Specifically, KEGG (Kanehisa and Goto, 2000) and Wikipathways (Wiki) (Kelder et al., 2012) pathway enrichment analysis were applied to each gene set in order to identify the top functionally enriched pathway categories related to the genes significantly differentially expressed in each contrast. Both KEGG and Wiki databases were used in an effort to generate a more comprehensive analysis. The KEGG system is recognized as one of the major pathway databases (Bauer-Mehren et al., 2009), whereby data is derived from published work. KEGG pathway content includes categories in metabolism, genetic information processing, organismal systems, environmental information processing, cellular processes, and drug development. The Wiki system is curated by the scientific community and serves as a complementary and enhancing source to the KEGG database (Bauer-Mehren et al., 2009). Finally, ANOVAs were performed on select genes of interest that were identified through the pathway analysis.

### Weighted gene correlational network analysis

Given that genes often operate in a coordinated manner to accomplish a physiological function, a more sophisticated approach utilizing Weighted Gene Correlational Network Analysis (WGCNA) was also performed. That is, in the absence of absolute differences in gene expression, the coexpression of genes may differ across experimental conditions. The WGCNA package within the R software program was used to perform this analysis. Following standard preprocessing and normalization of the data, a gene expression profile was available for each rat. Based on this expression profile, rats were clustered hierarchically within a dendogram based on Euclidian distance, or similarity between expression profiles. The dendogram was visualized to see how the physical traits (experimental conditions) related to the various clusters. Next, modules of highly coexpressed genes were identified and related to physical traits. Importantly, the genes within each module are more highly correlated with each other than to the rest of the transcriptome. Physical traits were categorized by experimental group (Sed.HC, Sed.Stress0, Sed.Stress2, Run.HC, Run.Stress0, Run.Stress2) and differences between groups (RunvsSed.HC, Stress0vsHC.Sed, Stress2vsHC.Sed, Stress0vsHC.Run, Stress2vsHC.Run, RunvsSed.Stress0, RunvsSed.Stress2). A correlation value and *p*-value associated with the strength of correlation was calculated for each module. These values are considered a representation of the correlation and correlational strength of the module to each physical trait. Modules with a correlational strength of *p* < 0.001 were targeted for further investigation by ANOVA. Following ANOVA analysis, modules that had statistically significant main effects of exercise, stress and/or an exercise by stress interaction were subjected to KEGG and Wiki analyses.

## Results

### Body weight and running distance

A repeated measures ANOVA was used to analyze body weights. Repeated measures ANOVA analysis revealed statistically significant main effects of time [*F*_(6, 252)_= 518.415; *p* < 0.0001] and exercise [*F*_(1, 42)_ = 7.759; *p* = 0.0080] and a reliable time by exercise interaction [*F*_(6, 252)_ = 2.634; *p* = 0.0171] on body weight. Running distance steadily increased over the course of the experiment from 5959.305 ± 382.081 m during week 1 to 19355.603 ± 2983.808 m during week 6 [*F*_(5, 115)_ = 18.870; *p* < 0.0001].

### Assessment of RNA and microarray Genechip quality

To verify microarray chip quality and mRNA integrity, boxplots were constructed that represented total mRNA expression for each rat. Visual inspection of boxplots revealed four outliers. One sample was previously identified with spectrophotometry analysis. The additional three outliers were dropped from further analysis. Final group totals were Sed_HC_ (*n* = 6), Sed_Stress0_ (*n* = 7), Sed_Stress2_ (*n* = 7), Run_HC_ (*n* = 8), Run_Stress0_ (*n* = 8), Run_Stress2_ (*n* = 8), for a total of (*n* = 44) rats.

### Differential gene expression analysis results

#### The effect of wheel running and/or exposure to stress on log fold changes in gene expression of ±1.1 in the DRN

**Figure 1:**
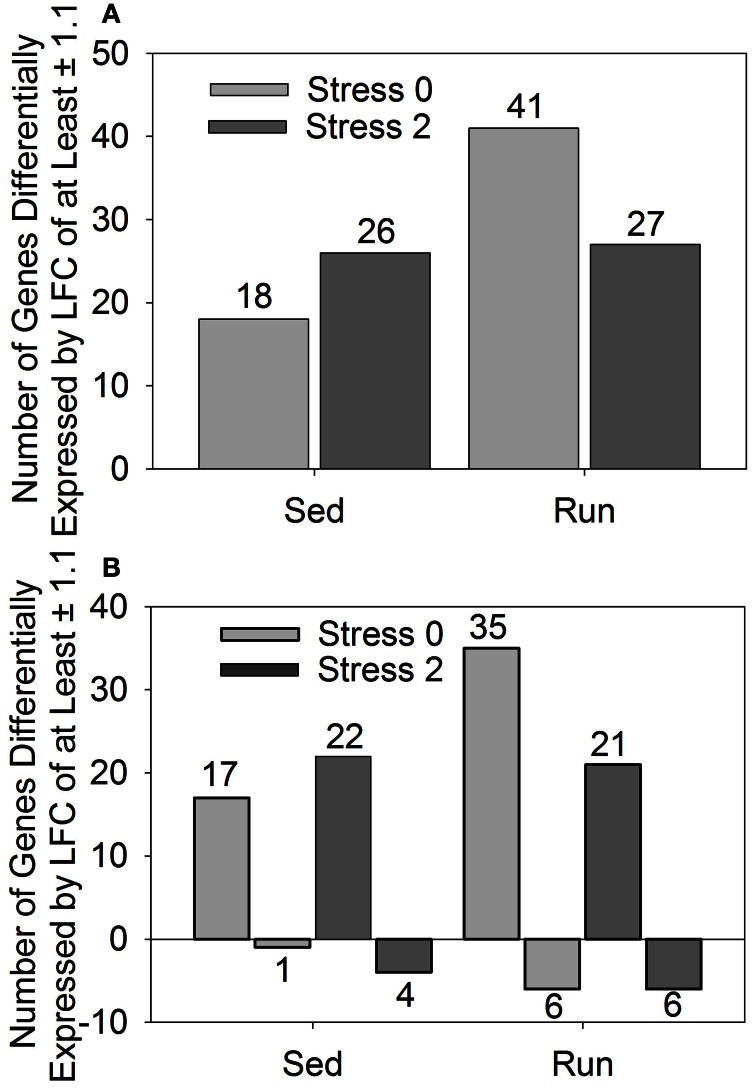
**Number of genes differentially expressed by a log fold change ≥ ± 1.1 in rats exposed to stress compared to home cage non-stressed controls. (A)** Total number of genes differentially expressed and **(B)** Number of genes differentially upregulated or downregulated.

In a conservative initial approach, genes differentially expressed by a LFC ≥ ±1.1 in response to exercise and/or stress were identified. Overall, relatively few genes had LFCs in expression ≥ ±1.1. The effect of stress on LFCs in gene expression ≥ ±1.1 is not different between sedentary and physically active rats immediately following or 2 h after stress exposure. When considering the effect of exercise, only one gene was statistically significantly altered. This gene was transthyretin, which was downregulated in physically active rats. Following stress exposure, transythretin remained downregulated in physically active rats immediately after, but not 2 h post stress. Compared to home cage controls, stress produced alterations in gene expression regardless of physical activity status at both time points. Figure 1 shows the effect of stress on total number of genes altered (A) and in which direction (B) by a LFC ≥ ±1.1 in sedentary rats and physically active rats immediately following and 2 h post stress relative to home cage non-stressed controls. Exposure to stress produced alterations in gene expression immediately following and 2 h after in both sedentary and physically active rats. A greater number of genes changed in physically active rats (41 and 27) than sedentary rats (18 and 26) for both time points (Figure 1A). All 18 genes that were differentially expressed immediately following stress in sedentary rats were also differentially expressed immediately following stress in physically active rats. Twenty-two of the twenty-six genes that were differentially expressed 2 h post stress in sedentary rats were also differentially expressed 2 h post stress in physically active rats. Two genes were differentially expressed exclusively in the physically active rats exposed to stress. These genes were CD180 and fos-like antigen 1, which were different at both time points. There were also two genes that were differentially expressed exclusively in sedentary rats exposed to stress compared to home cage non-stressed controls. These genes were only differentially expressed 2 h post stress and were fibronectin 1 and solute carrier organic anion transporter family, member 1c1.

Figure 1B shows the number of genes that were upregulated and downregulated by a LFC ≥ 1.1 in sedentary and physically active rats in response to stress. For all groups, a greater number of genes were differentially upregulated than downregulated in stressed rats compared to home cage non-stressed controls.

#### The effect of wheel running and/or exposure to stress on changes in gene expression of p < 0.05 in the DRN

**Figure 2:**
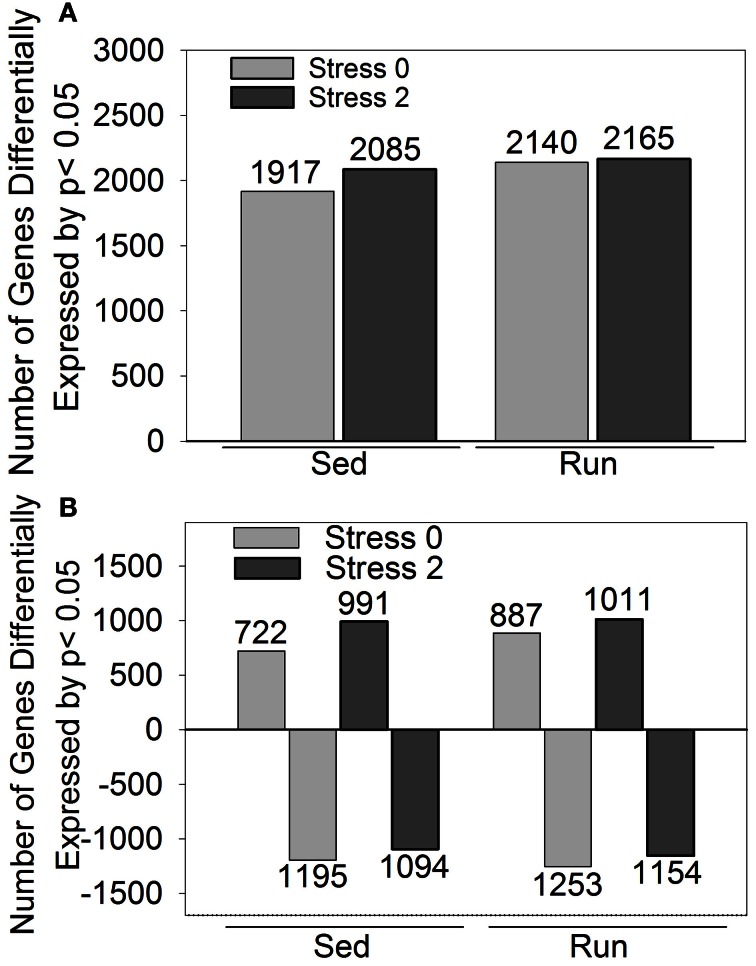
**Number of genes differentially expressed by *p* < 0.05 in rats exposed to stress compared to home cage non-stressed controls. (A)** Total number of genes differentially expressed. **(B)** Number of genes differentially upregulated or downregulated.

**Figure 3:**
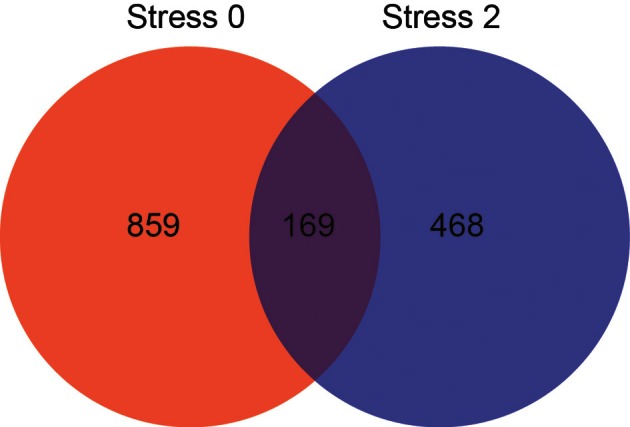
**Effect of physical activity status on stress-induced differential gene expression in the DRN by *p* < 0.05.** Number of genes differentially expressed immediately following (red) and 2 hours post (blue) stress, and genes differentially expressed at both time points (purple).

A second less stringent approach utilized *p*-values that were corrected for multiple comparisons to identify genes that were differentially expressed by *p* < 0.05 in response to exercise and/or stress. The effect of stress on differential gene expression is different depending on physical activity status. Differential expression of 1028 genes was observed immediately following stress and 637 genes were differentially expressed 2 h post stress in sedentary rats compared to physically active rats. (These results are detailed in Figure 3 and Table 3). Compared to sedentary rats, physically active rats had differential expression in 2350 genes (1290 upregulated, 1060 downregulated). Immediately following stress, differential expression of 634 genes (413 upregulated, 221 downregulated) was observed in physically active rats compared to sedentary rats. Two hours post stress, 997 genes (610 upregulated, 387 downregulated) were differentially expressed in physically active rats compared to sedentary rats. Compared to home cage non-stressed controls, stress produced changes in gene expression in both sedentary and physically active rats. Figure 2 shows the effect of stress on total number of genes altered (A) and in which direction (B) by *p* < 0.05 in sedentary rats and physically active rats immediately following and 2 h post stress relative to home cage non-stressed controls. Exposure to stress produced alterations in gene expression immediately following and 2 h after stress in both sedentary and physically active rats. For both time points, physically active rats exposed to stress had a greater number of genes differentially expressed relative to home cage non-stressed controls than sedentary rats exposed to stress (Figure 2A). For both sedentary and physically active rats, stress altered the expression of a greater number of genes 2 h after compared to immediately following stress. This difference was greater in sedentary rats.

Figure 2B indicates the number of genes that were upregulated and downregulated by *p* < 0.05 in sedentary and physically active rats in response to stress. For both sedentary and physically active rats, a greater number of genes were differentially upregulated 2 h after compared to immediately following stress. In contrast, the number of genes differentially downregulated was greater immediately following stress compared to 2 h after for both sedentary and physically active rats. For both time points, physically active rats had a greater number of genes upregulated than sedentary rats. Similarly, physically active rats had a greater number of genes downregulated than sedentary rats at both time points.

#### KEGG functionally enriched pathway categories related to genes differentially expressed in the DRN following 6 weeks of wheel running and/or exposure to stress

Genes that were differentially expressed at *p* < 0.05 across the various contrasts were imported into Web Gestaldt bioinformatics system and subjected to analysis with KEGG functional terms. Table 1 shows the top ten functionally enriched pathway categories, for each contrast, related to genes differentially expressed following 6 weeks of wheel running and/or exposure to stress. Gene count refers to the number of genes from the data sets that contribute to each functional category. The *p*-value represents the statistical significance of each functionally enriched category identified.

**Table 1:** **KEGG functionally enriched pathway categories generated from genes significantly differentially expressed at *p* < 0.05 in the DRN in response to exercise and/or stress**.

**Contrast**	**Gene count**	***P*-value**
**FUNCTIONALLY ENRICHED PATHWAY**		
**(Sed**_**Stress0**_ **vs. Sed**_**HC**_) **vs. (Run**_**Stress0**_ **vs. Run**_**HC**_)		
Olfactory transduction:04740	70	1.65e–13
Ribosome:03010	18	9.81e–12
Metabolic pathways:01100	69	1.48e–10
MAPK signaling pathway:04010	23	6.84e–07
Neuroactive ligand receptor interaction:04080	20	1.92e–05
Pathways in cancer:05200	23	2.34e–05
Endocytosis:04144	17	5.79e–05
TGF beta signaling pathway:04350	10	6.16e–05
ErbB signaling pathways:04012	10	0.0001
Arachidonic acid metabolism:00590	9	0.0010
**(Sed**_**Stress2**_ **vs. Sed**_**HC**_) **vs. (Run**_**Stress2**_ **vs. Run**_**HC**_)		
Olfactory transduction:04740	66	1.78e–21
Allograft rejection:05330	8	7.41e–06
Pathways in cancer:05200	18	1.16e–05
Autoimmune thyroid disease:05320	8	2.27e–05
Graft-vs.-host disease:05332	7	5.39e–05
C21-Steroid hormone metabolism:00140	4	4.44e–05
Androgen and estrogen metabolism:00150	6	3.77e–05
Type I diabetes mellitus:04940	7	0.0001
Non-small cell lung cancer:05223	6	0.0003
VEGF signaling pathway:04370	7	0.0003
**Run**_**HC**_ **vs. Sed**_**HC**_		
Metabolic pathways:01100	141	3.24e–17
Olfactory transduction:04740	115	4.84e–12
MAPK signaling pathway:04010	48	3.22e–12
Pathways in cancer:05200	53	2.28e–11
Ribosome:03010	23	6.46e–10
FC epsilon RI signaling pathway:04664	21	2.54e–09
Cell cycle:04110	27	3.83e–09
Long term depression:04730	19	5.84e–09
Gap junction:04540	21	1.65e–08
Vascular smooth muscle contraction:04270	24	4.09e–08
**Sed**_**Stress0**_ **vs. Sed**_**HC**_		
MAPK signaling pathway:04010	58	1.10e–21
Metabolic pathways:01100	104	2.33e–09
VEGF signaling pathway:04370	19	5.06e–09
Neurotropin signaling pathway:04722	25	9.26e–09
Pathways in cancer:05200	42	2.58e–08
Chronic myeloid leukemia:05220	17	4.77e–07
Toll-like receptor signaling pathway:04620	18	7.50e–07
GnRH signaling pathway:04912	16	1.93e–05
Leukocyte transendothelial migration:04670	18	2.29e–05
FC epsilon RI signaling pathways:04664	14	2.75e–05
**Sed_**Stress2**_ vs. Sed_**HC**_**		
MAPK signaling pathway:04010	56	9.54e–19
Pathways in cancer:05200	53	8.51e–13
Adipocytokine signaling pathway:04920	22	1.95e–12
Metabolic pathways:01100	116	3.16e–11
VEGF signaling pathway:04370	19	1.68e–08
Neuroactive ligand receptor interaction:04080	36	1.80e–07
Jak-STAT signaling pathway:04630	25	2.22e–07
Leukocyte transendothelial migration:04670	22	3.05e–07
Regulation of actin cytoskeleton:04810	31	4.89e–07
Toll-like receptor signaling pathway:04620	19	4.78e–07
**Run_**Stress0**_ vs. Run_**HC**_**		
MAPK signaling pathway:04010	63	2.05e–23
Metabolic pathways:01100	125	9.13e–14
Pathways in cancer:05200	50	5.84e–11
Cytokine-cytokine receptor interaction:04060	35	5.59e–10
Adipocytokine signaling pathway:04920	17	6.23e–08
Focal adhesion:04510	31	8.65e–08
P53 signaling pathway:04115	17	3.64e–07
Neuroactive ligand receptor interaction:04080	35	7.63e–07
Calcium signaling pathway:04020	28	1.25e–06
Neutrotrophin signaling pathway:04722	22	3.15e–06
**Run**_**Stress2**_ **vs. Run**_**HC**_		
Pathways in cancer:05200	74	3.25e–25
MAPK signaling pathway:04010	62	3.30e–22
Metabolic pathways:01100	127	8.15e–14
Jak-STAT signaling pathway:04630	35	1.15e–13
Neuroactive ligand receptor interaction:04080	44	4.00e–11
Chronic myeloid leukemia:05220	23	6.70e–11
Cytokine-cytokine receptor interaction:04060	37	6.25e–11
Focal adhesion:04510	35	8.43e–10
Prostate cancer:05215	23	1.01e–09
Pancreatic cancer:05212	19	1.20e–08
**Run**_**Stress0**_ **vs. Sed**_**Stress0**_		
Cytokine-cytokine receptor interaction:04060	12	7.82e–05
Pathways in cancer:05200	15	0.0003
Chemokine signaling pathway:04062	9	0.0018
MAPK signaling pathway:04010	11	0.0038
Toll-like receptor signaling pathway:04620	6	0.0038
Olfactory transduction:04740	28	0.0032
Apoptosis:04210	6	0.0047
Arachidonic acid metabolism:00590	5	0.0058
Jak-STAT signaling pathway:04630	7	0.0086
TGF beta signaling pathway:04350	5	0.0103
**Run_**Stress2**_ vs. Sed_**Stress2**_**		
Neuroactive ligand receptor interaction:04080	23	2.38e–07
Prostate cancer:05215	13	6.23e–07
Pathways in cancer:05200	24	4.38e–06
Ribosome:03010	11	1.22e–05
Focal adhesion:04510	16	4.16e–05
Regulation of actin cytoskeleton:04810	17	3.59e–05
Melanoma:05218	9	8.38e–05
Olfactory transduction:04740	44	0.0004
Cell adhesion molecule:04514	12	0.0006
Wnt signaling pathway:04310	11	0.0011

KEGG analysis revealed that genes that were differentially expressed between sedentary and physically active rats in response to stress were related to functional categories including metabolic pathways, mitogen-activated protein kinase (MAPK) signaling, neuroactive ligand receptor interaction, transforming growth factor-β (TGF-β) signaling, epidermal growth factor family of receptor tyrosine kinases (ErbB) signaling, and vascular endothelial growth factor (VEGF) signaling immediately following and or 2 h post stress.

Six weeks of wheel running modulated the expression of genes involved in physiological processes including metabolic activity, olfactory transduction, MAPK signaling, cell cycle, and long term depression.

Compared to home cage non-stressed controls, both sedentary and physically active rats exposed to stress had significant enrichment of functional categories related to MAPK signaling, metabolic pathways, adipocytokine signaling, and neuroactive ligand receptor interaction. Significant enrichment of functional categories including VEGF signaling and toll-like receptor signaling was exclusive to sedentary stressed rats compared to home cage non-stressed controls and occurred at both time points. Compared to home cage non-stressed controls, stress modulated the expression of genes involved in cytokine-cytokine receptor interaction exclusively in physically active rats at both time points.

A direct comparison of sedentary and physically active rats exposed to stress, revealed enrichment differences in functional categories related to cytokine-cytokine receptor interaction, chemokine signaling, MAPK signaling, toll-like receptor signaling, apoptosis, janus kinase-signal transducer and activator of transcription (Jak-Stat) signaling, TGF-β signaling, neuroactive ligand receptor interaction, cell adhesion molecules, and wingless-type mouse mammary tumor virus integration site (WNT) signaling either immediately following and/or 2 h post stress.

#### Wiki functionally enriched pathway categories related to genes differentially expressed in the DRN following 6 weeks of wheel running and/or exposure to stress

Genes that were differentially expressed at *p* < 0.05 across the various contrasts were imported into Web Gestaldt bioinformatics system and subjected to analysis with Wiki functional terms. Table 2 shows the top ten functionally enriched pathway categories, for each contrast, related to genes differentially expressed following 6 weeks of wheel running and/or exposure to stress. Gene count refers to the number of genes from the data sets that contribute to each functional category. The *p*-value represents the significance of each functionally enriched category identified.

**Table 2:** **Wiki functionally enriched pathway categories generated from genes significantly differentially expressed at *p* < 0.05 in the DRN in response to exercise and/or stress**.

**Contrast**	**Gene count**	***P*-value**
**FUNCTIONALLY ENRICHED PATHWAY**		
**(Sed**_**Stress0**_ **vs. Sed**_**HC**_) **vs. (Run**_**Stress0**_ **vs. Run**_**HC**_)		
Cytoplasmic ribosomal proteins:WP30	17	3.08e–09
MAPK signaling pathway:WP358	14	2.17e–05
IL-5 signaling pathway:WP44	9	4.29e–05
Insulin signaling:WP439	13	7.33e–05
B cell receptor signaling pathway:WP285	13	0.0001
Diurnally regulated genes with circadian orthologs:WP1306	6	0.0005
TGF beta receptor signaling pathway:WP362	11	0.0005
Adipogenesis:WP155	10	0.0008
Fas pathway and stress induction of HSP regulation:WP89	6	0.0007
IL-6 signaling pathway:WP135	9	0.0008
**(Sed**_**Stress2**_ **vs. Sed**_**HC**_) **vs. (Run**_**Stress2**_ **vs. Run**_**HC**_)		
Biosynthesis of aldosterone and cortisol:WP508	2	0.0038
Diurnally regulated genes with circadian orthologs:WP1306	4	0.0037
Steroid biosynthesis:WP66	2	0.0070
TNF alpha NF-kB signaling pathway:WP457	6	0.0579
GPCRs, class A rhodopsin-like:WP473	7	0.0485
Kit receptor signaling pathway:WP147	4	0.0206
Inflammatory response pathway:WP40	2	0.0666
Cytokines and inflammatory response:WP271	2	0.0494
Ovarian infertility genes:WP263	2	0.0535
Metapathway biotransformation:WP1286	5	0.0379
**Run**_**HC**_ **vs. Sed**_**HC**_		
MAPK signaling pathway:WP358	29	2.69e–09
EGFR1 signaling pathway:WP5	29	3.80e–08
TNF alpha NF-KB signaling pathway:WP457	30	3.17e–08
Insulin signaling:WP439	25	4.32e–07
Renin-angiotensin system:WP376	13	3.97e–07
Myometrial relaxation and contraction pathways:WP140	24	5.98e–07
Regulation of actin cytoskeleton:WP351	24	6.89e–07
G protein signaling pathways:WP73	18	1.14e–06
IL-5 signaling pathway:WP44	15	3.31e–06
B cell receptor signaling pathway:WP285	24	5.24e–06
**Sed**_**Stress0**_ **vs. Sed**_**HC**_		
MAPK signaling pathway:WP358	36	2.63e–16
Insulin signaling:WP439	31	1.25e–12
TGF beta receptor signaling pathway:WP362	25	2.96e–09
GPCRs, class A rhodopsin-like:WP473	32	4.84e–09
Adipogenesis:WP155	23	6.55e–09
EGFR1 signaling pathway:WP5	26	7.67e–08
IL-6 signaling pathway:WP135	19	1.53e–07
Toll-like receptor signaling pathway:WP1309	16	5.76e–07
IL-3 signaling pathway:WP319	18	5.39e–07
B cell receptor signaling pathway:WP285	23	1.21e–06
**Sed**_**Stress2**_ **vs. Sed**_**HC**_		
MAPK signaling pathway:WP358	38	6.26e–17
Adipogenesis:WP155	28	5.99e–12
EGFR1 signaling pathway:WP5	33	1.42e–11
B cell receptor signaling pathway:WP285	28	5.19e–09
Insulin signaling:WP439	27	4.64e–09
IL-3 signaling pathway:WP319	21	1.29e–08
IL-6 signaling pathway:WP135	21	1.93e–08
Toll-like receptor signaling pathway:WP1309	18	5.10e–08
Delta notch signaling pathway:WP199	18	5.10e–08
TNF alpha NF-KB signaling pathway:WP457	28	6.69e–08
**Run**_**Stress0**_ **vs. Run**_**HC**_		
MAPK signaling pathway:WP358	33	8.27e–13
Insulin signaling:WP439	31	1.27e–11
Adipogenesis:WP155	27	5.08e–11
EGFR1 signaling pathway:WP5	31	4.54e–10
Apoptosis mechanisms:WP284	21	1.50e–09
Apoptosis:WP1290	21	1.91e–09
Diurnally regulated genes with circadian orthologs:WP1306	13	6.73e–09
Cardiovascular signaling:WP590	14	3.15e–08
Toll-like receptor signaling pathway:WP1309	24	7.80e–08
T cell receptor signaling pathway:WP352	23	9.49e–08
**Run**_**Stress2**_ **vs. Run**_**HC**_		
MAPK signaling pathway:WP358	40	4.75e–18
Adipogenesis:WP155	33	1.25e–15
EGFR1 signaling pathway:WP5	39	2.01e–15
Insulin signaling:WP439	35	2.93e–14
B cell receptor signaling pathway:WP285	33	5.96e–12
IL-6 signaling pathway:WP135	26	5.32e–12
Delta notch signaling pathway:WP199	21	3.62e–10
TGF beta signaling pathways:WP505	17	6.00e–10
GPCRs, class A rhodopsin-like:WP473	34	5.30e–09
TGF beta receptor signaling pathway:WP362	26	6.09e–09
**Run**_**Stress0**_ **vs. Sed**_**Stress0**_		
Delta notch signaling pathway:WP199	8	2.30e–05
Kit receptor signaling pathway:WP147	7	6.36e–05
IL-5 signaling pathway:WP44	7	7.03e–05
IL-3 signaling pathway:WP319	7	0.0007
IL-6 signaling pathway:WP135	7	0.0007
TGF beta signaling pathways:WP505	5	0.0011
Toll-like receptor signaling pathway:WP1309	6	0.0012
Notch signaling pathway:WP517	4	0.0017
Endochondral ossification:WP1308	5	0.0017
Hedgehog signaling pathway:WP574	3	0.0026
**Run**_**Stress2**_ **vs. Sed**_**Stress2**_		
Adipogenesis:WP155	11	0.0001
GPCRs, class A rhodopsin-like:WP473	15	0.0002
B cell receptor signaling pathway:WP285	12	0.0004
Hypothetical network for drug addiction:WP1281	5	0.0007
IL-6 signaling pathway:WP135	9	0.0006
Regulation of actin cytoskeleton:WP351	11	0.0006
Calcium regulation in the cardiac cell:WP326	10	0.0009
Cytoplasmic ribosomal proteins:WP30	9	0.0017
Androgen receptor signaling pathway:WP68	9	0.0019
Myometrial relaxation and contraction pathways:WP140	10	0.0019

Wiki analysis revealed that genes that were differentially expressed between sedentary and physically active rats in response to stress were related to functional categories including metabolic pathways, MAPK signaling, adipogenesis, biosynthesis of aldosterone and cortisol, and diurnally regulated genes with circadian orthologs. In addition, various immune-associated categories were also identified including those related to the inflammatory and cytokine response as well as signaling pathways for interleukin-5 (IL-5), IL-6, B-cell receptor, TGF-β receptor, and TNF-α-nuclear factor kappa-light-chain-enhancer of activated B cells (NF-κB).

Six weeks of wheel running modulated the expression of genes involved in physiological processes related to signaling pathways for MAPK, epidermal growth factor receptor 1 (EGFR1), TNF-α-NF-κB, Insulin, G-protein, IL-5, and B-cell receptor. Compared to home cage non-stressed controls, both sedentary and physically active rats exposed to stress had enrichment of functional categories related to signaling pathways for MAPK, insulin, TGF-β receptor, IL-6, EGFR1, delta notch, and toll-like receptor. Other genes affected by stress were related to functional categories including G protein coupled receptors (GPCRs) and adipogenesis. Compared to home cage non-stressed controls, stress modulated the expression of genes involved in interleukin-3 (IL-3) signaling exclusively in sedentary rats at both time points. Significant enrichment of categories related to apoptosis, diurnally regulated genes with circadian orthologs, cardiovascular signaling, and T-cell receptor signaling was exclusive to physically active stressed rats compared to home cage non-stressed controls.

A direct comparison of sedentary and physically active rats exposed to stress, revealed significant differences in functional categories related to signaling pathways including delta notch, kit receptor, IL-3, IL-5, IL-6, TGF-β receptor, toll-like receptor, and B-cell receptor. Significant enrichment of functional categories related to adipogenesis and GPCRs was also observed.

#### The effect of stress on differential expression of genes in the DRN is different depending on physical activity status

The effect of stress on gene expression in the DRN is different depending on physical activity status. Figure 3 shows the number of genes changed by *p* < 0.05 that were differentially expressed between sedentary rats and physically active rats immediately following or 2 h post stress. Of these genes, 1028 were differentially expressed immediately following stress. Differential expression of 637 genes was observed 2 h after stress. Differential expression of 169 genes was observed in sedentary rats compared to physically active rats immediately following stress and differential expression of these genes was also present 2 h after stress.

Genes that were differentially expressed at both time points (*n* = 169) were subjected to KEGG pathway analysis (Table 3) in order to identify functionally enriched pathway categories related to these genes. Stress differentially impacted the expression of genes related to functional categories including olfactory transduction, metabolic pathways, MAPK signaling pathways, and VEGF signaling pathways in physically active compared to sedentary rats.

**Table 3:** **Functionally enriched KEGG and Wiki pathway categories related to genes differentially expressed in physically active and sedentary rats immediately following and 2 h post stress**.

**Pathway analysis**	**Gene count**	***P*-value**
**FUNCTIONALLY ENRICHED PATHWAY**		
**KEGG ENRICHMENT ANALYSIS**		
Olfactory transduction:04740	19	2.80en–08
Pathways in cancer:05200	8	0.0001
Metabolic pathways:01100	15	0.0001
Allograft rejection:05330	4	0.0001
MAPK signaling pathway:04010	7	0.0002
Autoimmune thyroid disease:05320	4	0.0003
VEGF signaling pathway:04370	4	0.0004
Intestinal immune network for IgA production:04672	3	0.0010
C21-Steroid hormone metabolism:00140	2	0.0014
Graft-vs. host disease:05332	3	0.0022
**WIKI ENRICHMENT ANALYSIS**		
Diurnally regulated genes with circadian orthologs:WP1306	3	0.0006
B cell receptor signaling pathway:WP285	4	0.0040
Cytokine and inflammatory response:WP271	2	0.0040
Tryptophan metabolism:WP270	2	0.0128
Regulation of actin cytoskeleton:WP351	3	0.0201
TGF beta signaling pathway:WP505	2	0.0195
MAPK signaling pathway:WP358	3	0.0238
Kit receptor signaling pathway:WP147	2	0.0309
IL-5 signaling pathway:WP44	2	0.0318
GPCRs, class A rhodopsin-like:WP473	3	0.0564

Genes that were differentially expressed at both time points (*n* = 169) were also subjected to Wiki pathway analysis (Table 3). Stress differentially impacted the expression of genes related to functional categories including diurnally regulated genes with circadian orthologs, tryptophan metabolism, and GPCRs in physically active compared to sedentary rats. Various immune-associated pathway categories were also identified such as cytokine and inflammatory response pathways as well as signaling pathways for B-cell receptor, TGF-β, and IL-5.

#### The effect of wheel running and/or exposure to stress on the expression of select genes related to functionally enriched wiki pathway categories

**Figure 4:**
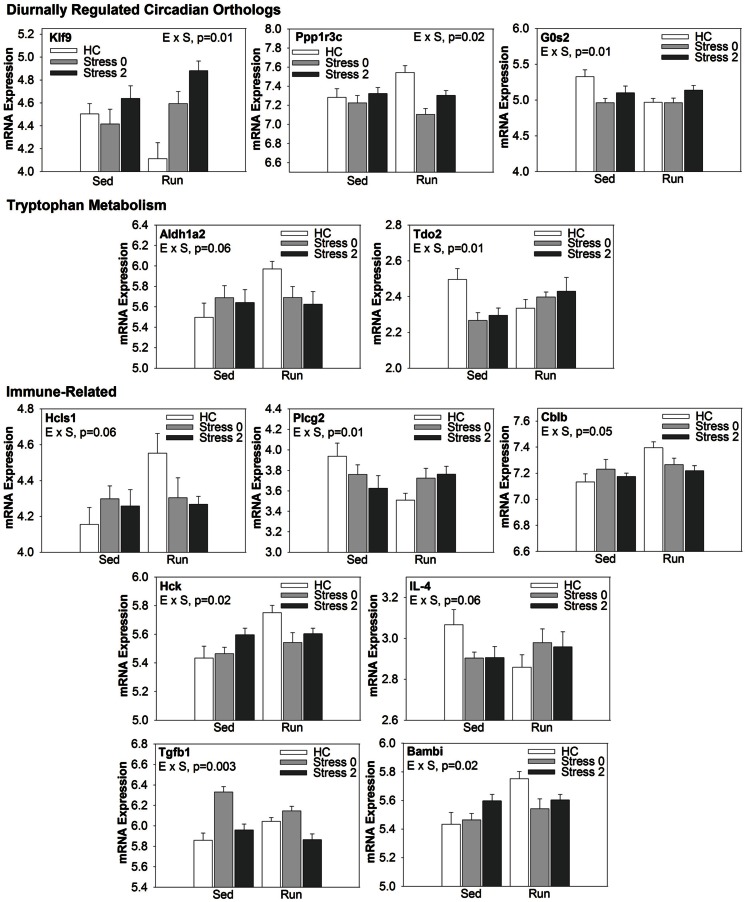
**Effect of exercise on stress-induced alterations in the expression of select genes in the DRN, organized by Wiki functional category**.

Select genes from Wiki functional pathway categories were targeted for analysis by ANOVA to reveal the main effect of wheel running, main effect of stress, and exercise x stress interaction on gene expression in the DRN immediately following and 2 h post stress. Table 4 shows the statistical results of the ANOVAs for each gene. Figure 4 displays the graphs for each ANOVA. Genes were selected from the following functional categories: diurnally regulated genes with circadian orthologs, tryptophan metabolism, and various immune-related pathways including B-cell receptor signaling, TGF-β signaling, IL-5 signaling, and cytokines and inflammatory response. Within the diurnally regulated genes with circadian orthologs category, genes selected for ANOVA were Kruppel-like factor 9 (*Klf9*), protein phosphate 1 (*Ppp1r3c*), and G0/G1 switch 2 (*G0s2*). Within the tryptophan metabolism category, genes selected for ANOVA were aldehyde dehydrogenase 1 family (*Aldh1a2*) and tryptophan 2,3-dioxygenase (*Tdo2*). Within the immune-related categories, genes selected for ANOVA included hematopoietic cell specific Lyn substrate 1 (*Hcls1*), phospholipase C, gamma 2 (*Plcg2*), Cas-Br-M ecotropic retroviral transforming sequence b (*Cblb*), hemopoietic cell kinase (*Hck*), interleukin 4 (*IL-4*), transforming growth factor, beta 1 (*TGF-β1*), and BMP and activin membrane-bound inhibitor (*Bambi*).

**Table 4:** **Effect of exercise on stress-induced alterations in the expression of select genes in the DRN**.

**FUNCTIONAL CATEGORY**			
***Gene***	**Exercise**	**Stress**	**Exercise × stress**
**DIURNALLY REGULATED W/CIRCADIAN ORTHOLOGS**			
*Klf9*	*F*_(1, 38)_ = 0.012, *p* = 0.915	*F*_(2, 38)_ = 7.842, *p* = 0.001	*F*_(2, 38)_ = 4.552, *p* = 0.016
*Ppp1r3c*	*F*_(1, 38)_ = 0.504, *p* = 0.482	*F*_(2, 38)_ = 6.551, *p* = 0.003	*F*_(2, 38)_ = 4.014, *p* = 0.026
*G0s2*	*F*_(1, 38)_ = 3.239, *p* = 0.079	*F*_(2, 38)_ = 3.813, *p* = 0.031	*F*_(2, 38)_ = 4.408, *p* = 0.019
**TRYPTOPHAN METABOLISM**			
*Aldh1a2*	*F*_(1, 38)_ = 2.713, *p* = 0.107	*F*_(2, 38)_ = 0.384, *p* = 0.683	*F*_(2, 38)_ = 2.891, *p* = 0.067
*Tdo2*	*F*_(1, 38)_ = 0.658, *p* = 0.422	*F*_(2, 38)_ = 1.243, *p* = 0.300	*F*_(2, 38)_ = 5.022, *p* = 0.011
**IMMUNE-RELATED**			
*Hcls1*	*F*_(1, 38)_ = 3.417, *p* = 0.072	*F*_(2, 38)_ = 0.494, *p* = 0.614	*F*_(2, 38)_ = 2.896, *p* = 0.067
*Plcg2*	*F*_(1, 38)_ = 1.898, *p* = 0.176	*F*_(2, 38)_ = 0.129, *p* = 0.879	*F*_(2, 38)_ = 4.352, *p* = 0.019
*Cblb*	*F*_(1, 38)_ = 7.493, *p* = 0.009	*F*_(2, 38)_ = 0.968, *p* = 0.389	*F*_(2, 38)_ = 3.179, *p* = 0.052
*Hck*	*F*_(1, 38)_ = 8.404, *p* = 0.006	*F*_(2, 38)_ = 1.858, *p* = 0.169	*F*_(2, 38)_ = 4.010, *p* = 0.026
*IL-4*	*F*_(1, 38)_ = 0.284, *p* = 0.597	*F*_(2, 38)_ = 0.121, *p* = 0.886	*F*_(2, 38)_ = 2.983, *p* = 0.062
*TGF-β 1*	*F*_(1, 38)_ = 0.528, *p* = 0.472	*F*_(2, 38)_ = 22.91, *p* < 0.0001	*F*_(2, 38)_ = 6.430, *p* = 0.003
*Bambi*	*F*_(1, 38)_ = 8.404, *p* = 0.006	*F*_(2, 38)_ = 1.858, *p* = 0.169	*F*_(2, 38)_ = 4.010, *p* = 0.026

### Weighted gene correlational network analysis

#### Hierarchical clustering of rats based on gene expression profiles

In order to construct modules of highly correlated genes that were related to exercise and/or stress exposure, a WGCNA was performed. Hierarchical clustering was used to categorize rats based on their individual expression profile of the genes within the transcriptome (17,170). First, a dendogram was constructed to cluster rats based on gene expression profile. Rats that were closer in distance within the dendogram were considered to have a more closely related gene expression profile. After the dendogram was constructed, clusters of the dendogram were related to physical traits whereby experimental condition (exercise and stress) were considered physical traits. With the exception of the outliers (*n* = 7), rats fell into two main categories, home cage non-stressed rats on the left and rats exposed to stress on the right. All groups within the stress cluster contained both sedentary and physically active rats with no visible grouping pattern by physical activity status. Within the home cage non-stressed control cluster, one stressed outlier was identified. Rats with the same physical activity status were clustered together within the non-stressed cluster.

#### Identification of modules of coexpressed genes in the DRN correlated to 6 weeks of wheel running and/or exposure to stress

Modules of highly coexpressed genes were identified that were also highly correlated (either positively or negatively) to exercise and/or stress. Eleven modules were derived from the transcriptome and were related to the various physical traits. Each module was assigned an arbitrary color. The number of genes contributing to each module was: yellow-199, blue-1077, purple-36, magenta-46, turquoise-3350, red-99, black-67, brown-373, green-153, pink-53, and grey-11,717. A correlation value and *p*-value associated with the correlational strength for each module-trait relationship was calculated. Modules of interest were identified based on statistical significance of the correlational strength (*p* < 0.001). A more stringent cutoff for statistical significance was used given the wide range of *p*-values (*p* = 3e−39 to *p* = 1.0). The grey module was excluded from analysis due to the large number of genes contributing to the module, and therefore, lack of specificity.

Of the 11 modules, 2 modules, the brown and black, were responsive to stress in both physically active and sedentary rats. The brown module was highly positively correlated to stress, indicating a strong increase in expression of genes within the brown module in response to stress. The physically active rats had a greater correlation value immediately following stress (0.98) compared to sedentary rats (0.83), suggesting that there was a more coordinated response among genes in the brown module in physically active rats. The black module was also highly positively correlated to stress, indicating a strong increase in expression of genes within the black module in response to stress. For both time points, physically active rats had a greater correlation value (0.96 and 0.83) compared to sedentary rats (0.8 and 0.72), suggesting that there was a more coordinated response among genes in the black module in physically active rats in response to stress.

Five additional modules were also identified. Genes within the blue, purple, and green modules were responsive to stress only in the sedentary rats. The blue module was negatively correlated (−0.5) with stress in sedentary rats, indicating a decrease in expression of genes within the blue module in response to stress. The purple module was negatively correlated (−0.5 and −0.65) with stress in sedentary rats immediately following and 2 h post stress, indicating a decrease in expression of genes within the purple module in response to stress. The green module was positively correlated (0.48) immediately following stress in sedentary rats, indicating an increase in expression of genes within the green module in response to stress.

Expression of genes within the purple and turquoise modules was associated with physical activity. The purple module was negatively correlated with physical activity (−0.48) indicating a decrease in expression of genes within the purple module in response to wheel running. The turquoise module was positively correlated with physical activity (0.53) indicating an increase in expression of genes within the turquoise module in response to wheel running.

Finally, the magenta module was positively correlated (0.53) with physically active rats 2 h post stress compared to sedentary rats 2 h post stress. This suggests that relative to sedentary rats, physically active rats have increased expression of genes within the magenta module 2 h following stress exposure.

#### The effect of wheel running and/or exposure to stress on modules of coexpressed genes in the DRN

**Figure 5:**
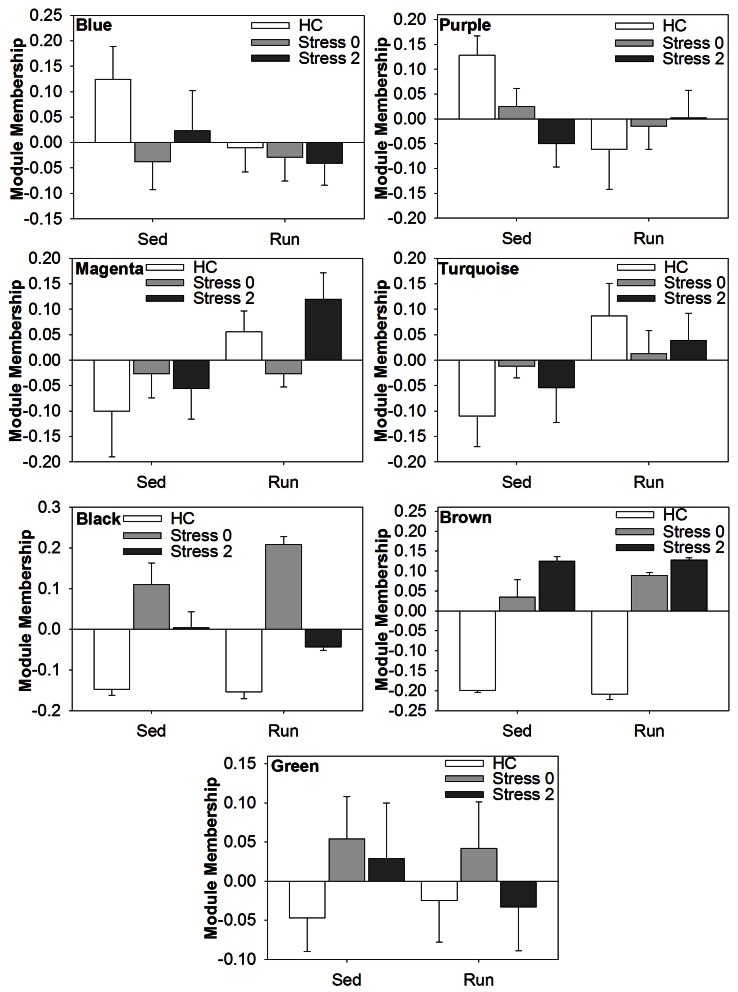
**Effect of exercise and/or stress on modules of coexpressed genes in the DRN**.

Modules of interest (correlational strength *p* < 0.001) were also subjected to analysis by ANOVA in order to determine the effect of exercise and/or stress on alterations in the coexpression of genes in the DRN. Figure 5 shows the graphs of the ANOVA analysis for each module. There were no statistically significant (*p* < 0.05) effects of exercise, stress, or an exercise by stress interaction in the blue, purple, or green module.

ANOVA analysis of the magenta module revealed a statistically significant main effect of exercise [*F*_(1, 38)_ = 6.588; *p* = 0.0143], but no significant main effect of stress or interaction. ANOVA analysis of the turquoise module also revealed a statistically significant main effect of exercise [*F*_(1, 38)_ = 5.495; *p* = 0.0244], but no significant main effect of stress or interaction. ANOVA analysis of the black module module revealed a statistically significant main effect of stress [*F*_(2, 38)_ = 56.872; *p* < 0.0001] and significant interaction [*F*_(2, 28)_ = 3.431; *p* = 0.0427], but no significant main effect of exercise. ANOVA analysis of the brown module revealed a statistically significant main effect of stress [*F*_(2, 38)_ = 168.838; *p* < 0.001], but no significant main effect of exercise or interaction.

#### KEGG functionally enriched pathway categories related to genes differentially coexpressed in the DRN following 6 weeks of wheel running and/or exposure to stress

For modules found to be statistically significant through ANOVA analysis, genes that contributed to each module were imported into Web Gestaldt bioinformatics system and subjected to analysis with KEGG functional terms. Table 5 shows the top ten functionally enriched pathway categories, for each module, related to the genes differentially coexpressed following 6 weeks of wheel running and/or exposure to stress. Gene count refers to the number of genes from the data sets that contribute to each functional category. The *p*-value represents the statistical significance of each functionally enriched category identified.

**Table 5:** **KEGG functionally enriched pathway categories generated from modules of genes correlated to exercise and/or stress**.

**Module**	**Gene count**	***P*-value**
**FUNCTIONALLY ENRICHED PATHWAY**		
**MAGENTA MODULE**		
Focal adhesion:04510	3	0.0014
Type II diabetes mellitus:04930	2	0.0015
Pancreatic cancer:05212	2	0.0031
Colorectal cancer:05210	2	0.0047
Pathways in cancer:05200	3	0.0061
Neurotropin signaling pathway:04722	2	0.0099
Insulin signaling pathway:04910	2	0.0099
Ubiquitin mediated proteolysis:04120	2	0.0091
Metabolic Pathways:01100	2	0.3704
**TURQUOISE MODULE**		
Metabolic Pathways:01100	231	3.92e–38
Ribosome:03010	43	7.08e–24
Ubiquitin mediated proteolysis:04120	45	9.21e–18
Axon guidance:04360	42	6.30e–15
Huntington's disease:05016	56	2.63e–13
Alzheimer's disease:05010	57	7.96e–13
Oxidative phosphorylation:00190	43	3.01e–12
Regulation of actin cytoskeleton:04810	51	3.72e–12
Spliceosome:03040	37	3.89e–12
Cell cycle:04110	37	5.05e–12
**BLACK MODULE**		
Cytokine-cytokine receptor interaction:04060	4	0.0005
Prion Diseases:05020	2	0.0020
Wnt signaling pathway:04310	3	0.0023
MAPK signaling pathway:04010	4	0.0014
Chemokine signaling pathway:04062	3	0.0039
Toll-like receptor signaling pathway:04620	2	0.0127
VEGF signaling pathway:04370	2	0.0087
GnRH signaling pathway:04912	2	0.0132
Apoptosis:04210	2	0.0137
Small cell lung cancer:05222	2	0.0127
**BROWN MODULE**		
MAPK signaling pathway:04010	23	3.94e–15
Cytokine-cytokine receptor interaction:04060	13	9.53e–08
Jak-Stat signaling pathway:04630	10	2.02e–06
Adipocytokine signaling pathway:04920	7	4.19e–06
Pathways in cancer:05200	14	6.26e–06
Hematopoietic cell lineage:04640	7	1.07e–05
P53 signaling pathway:04115	6	9.48e–05
Focal adhesion:04510	9	0.0002
Chronic myeloid leukemia:05220	6	0.0002
ErbB signaling pathway:04012	6	0.0002

KEGG analysis revealed that genes within the black module were related to functional categories including prion diseases, Wnt signaling, chemokine signaling, toll-like receptor signaling, VEGF signaling, gonadotropin-releasing hormone (GnRH) signaling, apoptosis, and small cell lung cancer. Genes within the brown module were related to functional categories including Jak-Stat signaling, adipocytokine signaling, pathways in cancer, hematopoietic cell lineage, p53 signaling, focal adhesion, chronic myeloid leukemia, and ErbB signaling. Both the black and brown modules contained genes that were related to functional categories of pathways involving cytokine-cytokine receptor interaction and MAPK signaling.

Genes within the magenta and turquoise modules were related to functional categories including metabolic pathways and ubiquitin mediated proteolysis. Refer to Table 5 for other functional categories related to genes within these modules.

#### Wiki functionally enriched pathway categories related to genes differentially coexpressed in the DRN following 6 weeks of wheel running and/or exposure to stress

For modules found to be statistically significant through ANOVA analysis, genes that contributed to each module were imported into Web Gestaldt bioinformatics system and subjected to analysis with Wiki functional terms. Table 6 shows the top ten functionally enriched pathway categories, for each module, related to the genes differentially coexpressed following 6 weeks of wheel running and/or exposure to stress. Gene count refers to the number of genes from the data sets that contribute to each functional category. The *p*-value represents the statistical significance of each functionally enriched category identified.

**Table 6:** **Wiki functionally enriched pathway categories generated from modules of genes correlated to exercise and/or stress**.

**Module**	**Gene count**	***P*-value**
**FUNCTIONALLY ENRICHED PATHWAY**		
**MAGENTA MODULE**		
Insulin signaling:WP439	3	0.0005
Apoptosis:WP1290	2	0.0039
IL-3 signaling pathway:WP319	2	0.0048
Apoptosis mechanisms:WP284	2	0.0038
**TURQUOISE MODULE**		
mRNA processing:WP529	41	2.45e–17
Electron transport chain:WP59	35	6.06e–17
TNF alpha NF-KB signaling pathway:WP457	47	2.77e–14
EGFR1 signaling pathway:WP5	42	6.39e–12
Regulation of actin cytoskeleton:WP351	37	2.25e–11
TGF beta receptor signaling pathway:WP362	35	1.04e–10
B cell receptor signaling pathway:WP285	38	1.96e–10
G protein signaling pathway:WP73	27	2.60e–10
Oxidative phosphorylation:WP1283	19	3.28e–09
Proteasome degradation:WP302	19	1.51e–08
**BLACK MODULE**		
Hypertrophy Model:WP442	3	4.38e–06
Insulin signaling:WP429	4	0.0001
GPCRs, class A rhodopsin-like:WP473	4	0.0005
Small ligand GPCRs:WP161	2	0.0004
Prostaglandin synthesis and regulation:WP303	2	0.0012
Myometrial relaxation and contraction pathways:WP140	3	0.0018
MAPK signaling pathway:WP358	3	0.0022
TGF beta receptor signaling pathway:WP362	3	0.0016
Diurnally regulated genes with circadian orthologs:WP1306	2	0.0022
Peptide GPCRs:WP131	2	0.0043
**BROWN MODULE**		
MAPK signaling pathway:WP358	15	1.29e–11
Adipogenesis:WP155	12	1.41e–09
Insulin signaling:WP429	9	1.04e–05
TGF beta receptor signaling pathway:WP362	8	3.95e–05
IL-6 signaling pathway:WP135	7	4.22e–05
Triacylglyercide synthesis:WP356	4	4.69e–05
ErbB signaling pathway:WP1299	5	7.64e–05
P38 MAPK signaling pathway:WP294	4	0.0002
GPCRs, class A rhodopsin-like:WP473	9	0.0002
Wnt signaling pathway and pluripotency:WP1288	6	0.0002
**GREEN MODULE**		
TNF alpha NF-KB signaling pathway:WP457	5	7.31e–05
Electron transport chain:WP59	3	0.0013
Androgen receptor signaling pathway:WP68	3	0.0029
Cytoplasmic ribosomal proteins:WP30	3	0.0028
Oxidative phosphorylation:WP1283	2	0.0070
Proteasome degradation:WP302	2	0.0081
G1 to S cell cycle control:WP348	2	0.0110
Wnt signaling pathway:WP375	2	0.0227

Wiki analysis revealed that genes within the brown module were related to functional categories including adipogenesis, IL-6 signaling, triacylglyceride synthesis, ErbB signaling, p38 MAPK signaling, and Wnt signaling and pluripotency. Genes within the black module were related to functional categories including hypertrophy model, small ligand GPCRs, prostaglandin synthesis and regulation, myometrial relaxation and contraction, diurnally regulated genes with circadian orthologs, and peptide GPCRs. Both the black and brown modules contained genes that were related to functional categories of pathways in insulin signaling, MAPK signaling, GPCRs of class A rhodopsin-like, and TGF-β receptor signaling.

For the magenta and turquoise modules, functional categories associated with immune pathways such as IL-3 signaling, insulin signaling, TGF-β receptor signaling, B-cell receptor signaling, and TNF-α-NF-κB signaling were identified.

## Discussion

### Overall themes

The mechanism by which exercise protects against the behavioral consequences of inescapable stress is unknown. The current data suggest that rats with 6 weeks of prior access to a running wheel have a different physiological response to stress, as measured by gene expression in the DRN, than sedentary rats. Here we report that (1) relative to home cage non-stressed controls, physically active rats have a greater number of genes differentially expressed in response to stress both immediately following and 2 h after stress exposure than sedentary rats (2) modules made up of genes that are highly coexpressed and responsive to stress operate in a more strongly coordinated manner in response to stress in physically active rats compared to sedentary rats (3) many of the stress-responsive genes within the DRN are known to be involved in various immune-related pathways, such as cytokine signaling and inflammatory processes.

These data demonstrate that in response to stress, physically active rats mount a more active response, at the level of mRNA transcription in the DRN. Relative to home cage non-stressed controls, physically active rats had a greater number of genes altered by a LFC ≥ ±1.1 and a greater number of genes significantly differentially altered by *p* < 0.05 than sedentary rats in response to stress. This is interesting because it suggests that the protective effect of exercise is not through a dampening of the stress response. Rather, physically active rats may mount a more robust response that functions, in concert, to protect the brain against the negative behavioral consequences of stress. Furthermore, physically active rats may respond to stress in a more efficient manner. This is demonstrated by the observation that modules made up of coexpressed genes that are highly stress-responsive, are more strongly upregulated in physically active rats in response to stress than sedentary rats. Given that these modules are highly upregulated in response to stress despite physical activity status, it is possible that the genes within these modules are expressed in order to protect the DRN from stress-induced damage. Physically active rats have a more strongly coordinated coexpression of these stress-responsive, “protective” genes and this more effective synchronization may be important for exercise-induced stress resistance.

Interestingly, many genes within the DRN that are altered in response to stress are involved in immune-related signaling processes including the signaling of proteins (MAPK) involved in the stimulation of proinflammatory factors, immune cell receptors (toll-like, B cell, T cell), cytokines (IL-3, IL-4, IL-5, IL-6, TGF-β, TNF-α) and cytokine receptors (TGF-β1), chemokines, and regulatory pathways involved in the immune response to infection (NF-κB). These various immune-related functional categories were identified in both differential expression and WGCNA analysis of genes and in both KEGG and Wiki pathway databases. It is important to note that identification of specific functional pathways does not necessarily imply that such processes are occurring within the DRN in response to exercise and/or stress. Rather, pathway identification is a means of organizing the thousands of genes that are significantly differentially expressed or coexpressed in response to exercise and/or stress, by functional relationships. Genes known to be involved in B cell receptor signaling, for example, were significantly altered in the DRN in response to stress. However, B cells are only present at very low levels in a healthy brain (Anthony et al., 2003). Thus, these genes are likely performing other functions. Additionally, it is likely that the immune-associated functional categories of genes within the KEGG and Wiki pathway databases were generated from data based on peripheral immune processes, and therefore, may not be an accurate representation of immune functions within the brain. However, given the evidence that physical activity has anti-inflammatory effects peripherally that confer protection against chronic disease (Gleeson et al., 2011), elucidation of the nature of the stress-induced inflammatory response in the DRN of physically active rats is of prime importance. It is possible that exercise confers protection by dampening the stress-induced inflammatory cascade in the brain.

Overall, these data suggest that at the level of mRNA transcription in the DRN, there is a colossal response to stress. A history of physical activity, changes, but does not necessarily dampen this response. Furthermore, there is evidence of stress-induced induction of inflammatory processes, though it is not clear whether there is an overall pro-inflammatory, anti-inflammatory, or balanced response. Additionally, it is not apparent if the overall inflammatory response is different depending on physical activity status. Regardless, these data provide evidence for a stress-induced inflammatory response originating in a region of the brain implicated in stress-related mood disorders, and exemplify the importance of investigating novel theories, such as the cytokine-induced hypothesis of depression.

### Novel targets of exercise-induced stress resistance

The goal of this experiment was to identify novel gene targets of exercise-induced stress resistance. The “novel” component was considered to be of particular importance and therefore, the data were analyzed without the guidance of an a priori hypothesis. Differential gene expression analysis was employed to narrow the transcriptome of 17,170 genes to those most likely involved in stress resistance. Specifically, contrasts were made between experimental groups to identify genes that were significantly differentially expressed by *p* < 0.05. The contrasts considered to be the most important for revealing exercise-induced stress resistance were the contrasts that revealed genes differentially expressed due to an exercise by stress interaction. More specifically, we were interested in identifying genes that were differentially expressed at *p* < 0.05 in response to stress depending on the physical activity status of the rat. The contrasts that provided this information were [(Sed_Stress0_ v. Sed_HC_) v. (Run_Stress0_ v. Run_HC_)] and [(Sed_Stress2_ v. Sed_HC_) v. (Run_Stress2_ v. Run_HC_)]. The first contrast generated a list of 1028 genes that were differentially expressed immediately following stress in physically active rats compared to sedentary rats. The second contrast generated a list of 637 genes that were differentially expressed 2 h following stress in physically active rats compared to sedentary rats. Given the large number of genes that were altered, an additional constraint was placed on the data. Only those genes that were differentially expressed at both time points were considered (*n* = 169). We reasoned that mRNA present immediately following stress and 2 h following stress was more likely to be translated into protein. The 169 genes that were differentially expressed in response to stress in physically active rats compared to sedentary rats were subjected to both KEGG and Wiki analysis, in order to organize the list of genes by functional categories. It is important to note that of the 169 genes, only 69 genes were fitted to KEGG specific functional categories, and only 26 genes were fitted to Wiki specific functional categories. Thus, it is possible that important genes were lost due to the limitations inherent in classifying genes into categories. From an a priori perspective, visual inspection of the genes not assigned to a KEGG or Wiki functional category did not reveal any compelling genes, *per se*. We chose to focus on genes classified by the Wiki database because the functional categories seemed to be tailored to more specific processes (TGF-β signaling and tryptophan metabolism pathways) compared to KEGG identified processes (metabolic pathways and pathways in cancer). From the Wiki categories, 12 genes were selected that were related to functional categories including diurnally regulated genes, tryptophan metabolism, and inflammatory-related processes. These categories were specifically chosen because the circadian system, serotonergic circuits, and inflammation have all been implicated in having a role in mood disorders. It should be noted that of the 12 genes selected from Wiki functional categories, 6 of these genes were also present in the KEGG classification of functional categories.

ANOVA analysis was performed in order to assess the differential effect of stress-induced changes within these genes in physically active compared to sedentary rats. Table 7 provides a brief description of the function associated with the protein that each gene encodes (Safran et al., 2010).

**Table 7:** **Functional role of proteins encoded by genes of interest**.

**FUNCTIONAL CATEGORY**	
***Gene***	**Protein product: function**
**DIURNALLY REGULATED W/CIRCADIAN ORTHOLOGS**	
*Klf9*	Transcription factor: can inhibit or activate transcription
*Ppp1r3c*	Enzyme: participates in variety of cellular processes by reversible protein phosphorylation
*G0s2*	Not clear: potential oncogene and regulator of latent HIV
**TRYPTOPHAN METABOLISM**	
*Aldh1a2*	Enzyme: catalyzes the synthesis of retinoic acid from retinaldehyde
*Tdo2*	Enzyme: catalyzes 1st and rate-limiting step in a major pathway of tryptophan metabolism, L-tryptophan > n-formyl kynurenine
**IMMUNE-RELATED**	
*Hcls1*	Substrate: role in antigen receptor signaling, potential role in regulation of gene expression
*Plcg2*	Enzyme: catalyze hydrolysis of phospholipids
*Cblb*	Ligase: negatively regulates T-cell and B-cell receptors
*Hck*	Enzyme: may help couple Fc receptor to activation of respiratory burst, potential role in neutrophil migration and degranulation of neutrophils
*IL-4*	Cytokine: forms a gene cluster w/ IL-3, IL-5, IL-13 on chromosome 5q, costimulator of DNA synthesis, induces expression of MHC II on B-cells
*TGF-β1*	Cytokine: controls proliferation, differentiation, regulation of other growth factors
*Bambi*	Receptor: related to type 1 receptors of TGF-β family

Of particular interest are *Tdo2* and *TGF-β 1* (Figure 4). Tryptophan is the precursor to 5-HT and therefore, serves as a regulator of 5-HT synthesis. The *Tdo2* gene encodes a major enzyme, tryptophan 2,3 dioxygenase (TDO), involved in tryptophan metabolism. TDO degrades tryptophan along the kynurenine pathway (for review, see Efimov et al., 2011). Interestingly, in rats exposed to stress, *Tdo2* mRNA expression in the DRN is differentially altered depending on physical activity status [ExS, *F*_(2, 38)_ = 5.022; *p* = 0.0116]. Fisher's PLSD post-hoc analyses revealed that exercise decreased baseline levels of *Tdo2* and there was no effect of stress on *Tdo2* expression in physically active rats. In contrast, sedentary rats had decreased *Tdo2* expression immediately following and 2 h post stress. Stress-induced decreases in *Tdo2* may lead to a reduction in TDO levels and decreased ability of rats to degrade tryptophan. This could contribute to the increased levels of 5-HT in the DRN following inescapable stress. Wheel running blocks stress-induced decreases in *Tdo2* mRNA expression, and thus, may prevent stress-induced decreases in TDO and the reduction of tryptophan metabolism in the DRN.

*TGF-β1* was an additional gene of interest. The *TGF-β1* gene encodes for the cytokine, TGF-β1. TGF-β1 is a regulator of T cells, and is typically considered to have anti-inflammatory properties. TGF-β1 has also been investigated in the context of depression. Kim et al. (2007) found increased levels of TGF-β1 in the plasma of patient's with major depression disorder and 6 weeks of treatment with antidepressants significantly decreased levels of TGF-β1. Our data suggests that *TGF-β1* mRNA in the DRN is particularly sensitive to stress (*p* < 0.0001) and differentially altered depending on physical activity status [ExS, *F*_(2, 38)_ = 6.430; *p* = 0.0039]. Fisher's PLSD post-hoc analysis revealed that exercise increased baseline levels of *TGF-β1* mRNA. There was a decrease in *TGF-β1* mRNA expression in physically active rats 2 h post stress. In contrast, sedentary rats had a large increase in *TGF-β1* immediately following stress. The significance of the impedance effect of exercise on stress-induced increases in *TGF-β1* mRNA levels in the DRN is unclear. Sedentary rats may have a more pro-inflammatory response to stress, and therefore, the increase in *TGF-β1* mRNA may occur in order to subdue pro-inflammatory responses. On the other hand, 5-HT treatment has been shown to increase *TGF-β1* mRNA expression in mesangial cells (Grewal et al., 1999) and therefore, increased *TGF-β1* mRNA expression in DRN cells may be due to stress-induced increases in 5-HT levels in sedentary rats. However, it is important to consider that the functional role of TGF-β1 may be different in the brain compared to peripheral tissue. Given the association of TGF-β1 with depression and antidepressant treatment, further examination of TGF-β1 is warranted.

It is important to consider that in the context of this particular analysis, the identification of novel targets of exercise-induced stress resistance was restricted to those genes that were differentially expressed due to the interaction between exercise and stress. However, it is possible that the mechanism by which exercise confers protection is not opposite of the mechanism by which stress produces negative consequences. Exercise-induced stress resistance could occur through a non-stress responsive route. Furthermore, identification of stress-resistant genes relied solely on the genes being differentially expressed in physically active rats compared to sedentary rats exposed to stress. However, genes often work in coordinated manners to carry out physiological functions. In the absence of absolute differences in gene expression, differences in the coexpression of gene networks in response to stress may underlie the protective effect of exercise. Identification of hub genes critically important to the modules detected with WGCNA will address this possibility, and may lead to the identification of additional targets. (Identification of hub genes is discussed in greater detail in Future Directions.)

### Advantages and limitations of microarray analysis

The greatest advantage of microarray analysis is that it enables the simultaneous exploration of the expression of thousands of genes. Therefore, it is particularly useful in studying complex processes, such as the stress response, whereby thousands of genes are affected. When used in conjunction with laser capture microarray technology, microarray has the potential to yield whole genome expression data about an organism's response to an environmental manipulation, such as exposure to stress or voluntary exercise, in a specific region of the brain or cell type within that region. It is important to point out that microarray analysis only provides information at the level of mRNA transcription, which is not necessarily indicative of protein production. Nevertheless, transcription initiation is the most widely used means of gene regulation in eukaryotes (Cox et al., 2012), and using an exploratory approach, microarray experiments serve as an important starting screen for the identification of novel targets that can be analyzed in greater detail with other techniques (Coppola, 2011).

**Figure 6:**
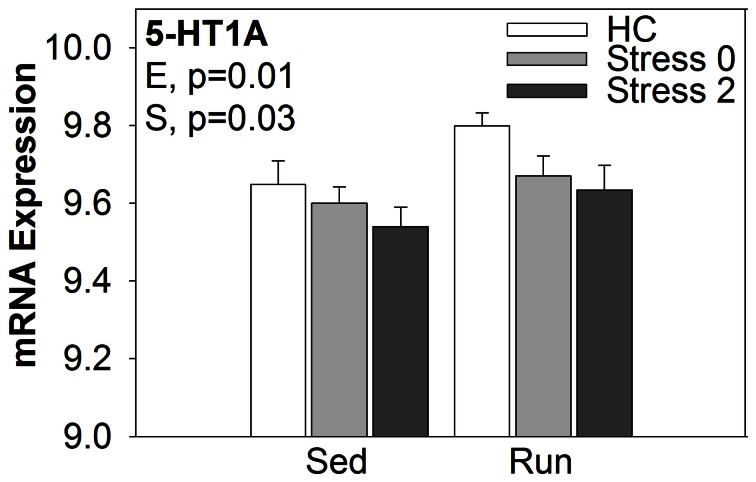
**mRNA expression of 5-HT1A in the DRN**.

The exploratory approach to microarray data analysis, however, is not without its limitations. This approach relies solely on statistical significance to identify often thousands of genes that in turn, must be organized in such a way that allows for interpretation. The organization of statistically significant genes, usually by functional categories derived from bioinformatics databases, may fail to identify genes that play a crucial role in the regulation of a given process. The 5HT_1*A*_ receptor, for example, is thought to be critically involved in the mechanism by which inescapable stress produces sensitization of DRN 5-HT neurons (Rozeske et al., 2011). Using in-situ hybridization, our lab has previously shown that wheel running increases 5-HT_1*A*_ mRNA expression in the DRN (Greenwood et al., 2003, 2005). An exploratory analysis of the current dataset did not identify 5-HT_1*A*_ as a target of interest. However, using an a priori guided approach, ANOVA analysis of 5-HT_1*A*_ mRNA in the DRN revealed significant main effects of exercise [*F*_(1, 38)_ = 6.250; *p* = 0.0169] and stress [*F*_(2, 38)_ = 3.538; *p* = 0.0390], but no significant interaction (Figure 6). Our data are consistent with the previous findings that 6 weeks of wheel running increases 5-HT_1*A*_ mRNA expression in the DRN (Greenwood et al., 2003). We also replicated our previous report that exposure to stress decreases 5-HT_1*A*_ mRNA expression in the DRN (Greenwood and Fleshner, 2011).

Overall, both the advantages and limitations of microarray technology are the product of the colossal dataset that a microarray experiment yields. Organization of the data is required so that statistically significant differences observed in thousands of genes can be focused into more manageable and interpretable gene sets. This process of data distillation, however, is not without a price and in the process, information may be lost. Given this reality, it is of paramount importance that multiple statistical and analytical strategies are executed. The traditional analysis of differential expression should be used in addition to the more sophisticated analysis of coexpression with WGCNA. Although both approaches return long lists of genes that must be further mined, each method assesses different regulatory processes. Finally, when identifying functional categories related to genes of interest, multiple bioinformatics databases should be explored. Inconsistencies in the information returned by bioinformatics databases (Soh et al., 2010) are pervasive, and using more than one database may provide more comprehensive results.

### Future directions

The overwhelming amount of data obtained in microarray experiments can be a challenge to manage, however, the results are powerful and provide researchers with a wealth of novel processes and genes to explore. To identify additional targets of exercise-induced stress resistance, hub genes should be identified within the modules of highly coexpressed genes detected by the WGCNA. Research suggests that a gene's position within a given network of genes, or module, is indicative of its functional significance to that module (Miller et al., 2008). More centralized genes, termed hub genes, are more likely to have critical roles in cellular function than peripheral genes (Miller et al., 2008). Therefore, centralized hub genes within the brown module may play a significant role in the effect of stress, while centralized hub genes within the black module (which was differentially altered in response to stress depending on physical activity status), may play a significant role in the protective effect of exercise against stress. Identification of hub genes within the brown and black modules could lead to the identification of additional therapeutic targets.

Further analysis of significantly regulated genes with assignment of cell type may also add richly to the dataset. More specifically, a database containing information on the genes enriched in a given cell type, such as neurons, astrocytes, and oligodendrocytes, can be used to assign cell type specificity to significantly differentially expressed or coexpressed genes (Cahoy et al., 2008). Given there was consistent evidence for inflammatory-related genes being upregulated in response to stress in the DRN, it may be insightful to identify the specific cell-type and source of this inflammatory-related mRNA expression.

Future experiments should include validating the novel targets identified with microarray analysis with PCR and/or in-situ hybridization. In-situ hybridization, in particular, could provide information on the anatomical specificity of the observed differences in mRNA expression in response to exercise and/or stress within the DRN. Additionally, novel gene targets of exercise-induced stress resistance should be tested by means of behavioral pharmacology. That is manipulation of the proteins encoded by these genes with specific agonists or antagonists in the context of inescapable stress exposure and/or exercise may reveal information on the therapeutic potential of these genes. Delivery of pharmacological agonsits of targets of upregulated “stress-resistance genes” to sedentary rats, for example, would be expected to confer protection against stress-induced behaviors in these rats in the absence of exercise.

Androgens and circadian regulationare additional topics that may warrant further investigation for having a role in the mechanisms by which exercise produces stress resistance. Our data suggest that pathways of genes related to androgen receptor signaling and diurnal regulation are differentially expressed in the DRN in physically active compared to sedentary rats following stress. Androgens promote neurogenesis (Spiritzer and Galea, 2007) and enhance cognitive function (Edinger and Frye, 2007). A recent study reported that mild exercise increased androgen synthesis in the hippocampus (Okamoto et al., 2012). It is possible that wheel running also increases androgen synthesis in the DRN and provides stress-resistance through an androgen-mediated pathway. Additionally, disruption of the circadian rhythm has been implicated in mood disorders. Interestingly, stimulation of the DRN triggers the release of 5-HT by the suprachiasmatic nucleus (SCN) (Dudley et al., 1999), the brain region responsible for the generation of circadian rhythms (Rusak and Zucker, 1979), and DRN stimulation induces circadian phase-resetting (Glass et al., 2000). Constraint of DRN 5-HT neurons in physically active rats could block stress-induced alterations in SCN output and provide stress resistance through the prevention of circadian rhythm disruption.

## Conclusions

In conclusion, when the data are organized effectively, microarray experiments have the ability to yield a rich amount of information on the molecular activities underlying physiological processes. When used in combination with laser capture microdissection, this information can be obtained from a specific region or cell type within an organism. Thus microarray technology is particularly useful in studying the neurobiological mechanisms underlying the complex pathophysiology of stress-related mood disorders. This experiment was designed to reveal novel targets by which exercise produces resistance to stress-related mood disorders, specifically within the DRN, using microarray and laser capture microdissection technology. The current data reveal evidence for different profiles of gene expression in the DRN of physically active rats exposed to stress compared to sedentary rats exposed to stress. Physically active rats have a more active and more strongly coordinated response to stress than sedentary rats. Specifically, Tdo2, a gene encoding an enzyme involved in tryptophan metabolism, may have a role in the mechanism by which exercise protects against the behavioral consequences of inescapable stress. In addition, an inflammatory-related gene encoding for the cytokine TGF-ß1 was particularly responsive to stress and this response was different depending on physical activity status. Overall, an inflammatory theme was revealed consistently across multiple analyses, suggesting a large effect of stress on inflammatory-related processes in cells of the DRN. The consequence of stress-induced inflammatory processes in the DRN should be further investigated.

### Conflict of interest statement

The authors declare that the research was conducted in the absence of any commercial or financial relationships that could be construed as a potential conflict of interest.

## References

[B1] Anthony I. C. Crawford D. H. Bell J. E. (2003). B lymphocytes in the normal brain: contrasts with HIV-associated lymphoid infiltrates and lymphomas. Brain 126, 1058–1067 10.1093/brain/awg118 12690046

[B2] Bauer-Mehren A. Furlong L. I. Sanz F. (2009). Pathway databases and tools for their exploitation: benefits, current limitations and challenges. Mol. Syst. Biol. 5:290 10.1038/msb.2009.47 19638971 PMC2724977

[B3] Belin M. F. Aguera M. Tappaz M. McRae-Degueurce A. Bobillier P. Pujol J. F. (1979). GABA-accumulating neurons in the nucleus raphe dorsalis and periaqueductal gray in the rat: a biochemical and radioautographic study. Brain Res. 170, 279–297 10.1016/0006-8993(79)90107-0 466412

[B4] Cahoy J. D. Emery B. Kaushal A. Foo L. C. Zamanian J. L. Christopherson K. S. (2008). A transcriptome database for astrocytes, neurons, and oligodendrocytes: a new resource for understanding brain development and function. J. Neurosci. 28, 264–278 10.1523/JNEUROSCI.4178-07.2008 18171944 PMC6671143

[B5] Casanovas J. M. Lésourd M. Artigas F. (1997). The effect of the selective 5-HT_1A_ agonists alnespirone (S-20499) and 8-OH-DPAT on extracellular 5-hydroxytryptamine in different regions of rat brain. Br. J. Pharmacol. 122, 733–741 10.1038/sj.bjp.0701420 9375971 PMC1564978

[B6] Celada P. Siuciak J. A. Tran T. M. Altar C. A. Tepper J. M. (1996). Local infusion of brain-derived neurotrophic factor modifies the firing pattern of dorsal raphé serotonergic neurons. Brain Res. 712, 293–298 10.1016/0006-8993(95)01469-1 8814905

[B7] Chaouloff F. (1993). Physiopharmacological interactions between stress hormones and central serotonergic systems. Brain Res. Rev. 18, 1–32 8467346 10.1016/0165-0173(93)90005-k

[B8] Christianson J. P. Paul E. D. Irani M. Thompson B. M. Kubala K. H. Yirmiya R. (2008). The role of prior stressor controllability and the dorsal raphé nucleus in sucrose preference and social exploration. Behav. Brain Res. 193, 87–93 10.1016/j.bbr.2008.04.024 18554730 PMC2583404

[B9] Commons K. G. Beck S. G. Bey V. W. (2005). Two populations of glutamatergic axons in the rat dorsal raphe nucleus defined by the vesicular glutamate transporters 1 and 2. Eur. J. Neurosci. 21, 1577–1586 10.1111/j.1460-9568.2005.03991.x 15845085 PMC2831872

[B10] Coppola G. (2011). Designing, performing, and interpreting a microarray-based gene expression study. Methods Mol. Bio. 793, 417–439 10.1007/978-1-61779-328-8_28 21913117

[B11] Cox M. M. Doudna J. A. O'Donnell M. (2012). Molecular Biology Principles and Practice. New York, NY: W. H. Freeman and Company

[B12] Cullinan W. E. Herman J. P. Battaglia D. F. Akil H. Watson S. J. (1995). Pattern and time course of immediate early gene expression in rat brain following acute stress. Neuroscience 64, 477–505 10.1016/0306-4522(94)00355-9 7700534

[B13] Cunningham E. T. De Souza E. B. (1993). Interleukin 1 receptors in the brain and endocrine tissues. Immunol. Today 14, 171–176 8499077 10.1016/0167-5699(93)90281-o

[B14] Day H. E. Curran E. J. Watson S. J. Akil H. (1999). Distinct neurochemical populations in the rat central nucleus of the amygdala and bed nucleus of the stria terminalis: evidence for their selective activation by interleukin-1beta. J. Comp. Neurol. 413, 113–128 10.1002/(SICI)1096-9861(19991011)413:1<113::AID-CNE8>3.0.CO;2-B 10464374

[B15] Day H. E. Greenwood B. N. Hammack S. E. Watkins L. R. Fleshner M. Maier S. F. (2004). Differential expression of 5HT-_1A_, alpha 1b adrenergic, CRF-R1, and CRF-R2 receptor mRNA in serotonergic, gamma-aminobutyric acidergic, and catecholaminergic cells of the rat dorsal raphe nucleus. J. Comp. Neurol. 474, 364–378 10.1002/cne.20138 15174080 PMC2430888

[B16] de Quidt M. E. Emson P. C. (1986). Distribution of neuropeptide Y-like immunoreactivity in the rat central nervous system–II. Immunohistochemical analysis. Neuroscience 18, 545–618 10.1016/0306-4522(86)90057-6 3755809

[B17] Drugan R. C. Ryan S. M. Minor T. R. Maier S. F. (1984). Librium prevents the analgesia and shuttlebox escape deficit typically observed following inescapable shock. Pharmacol. Biochem. Behav. 21, 749–754 6542677 10.1016/s0091-3057(84)80014-3

[B18] Dudley T. E. Dinardo L. A. Glass J. D. (1999). *In vivo* assessment of the midbrain raphe nuclear regulation of serotonin release in the hamster suprachiasmatic nucleus. J. Neurophysiol. 81, 1469–1477 10200183 10.1152/jn.1999.81.4.1469

[B19] Edinger K. L. Frye C. A. (2007). Androgens' performance-enhancing effects in the inhibitory avoidance and water maze task involve actions at intracellular androgen receptors in the dorsal hippocampus. Neurobiol. Learn. Mem. 87, 201–208 10.1016/j.nlm.2006.08.008 17029870

[B20] Efimov I. Basran J. Thackray S. J. Handa S. Mowat C. G. Raven E. L. (2011). Structure and reaction mechanism in the heme dioxygenases. Biochemistry 50, 2717–2724 10.1021/bi101732n 21361337 PMC3092302

[B21] Ferré S. Cortés R. Artigas F. (1994). Dopaminergic regulation of the serotonergic raphe-striatal pathway: microdialysis studies in freely moving rats. J. Neurosci. 14, 4839–4846 7519257 10.1523/JNEUROSCI.14-08-04839.1994 PMC6577199

[B22] Fleshner M. Deak T. Spencer R. L. Laudenslager M. L. Watkins L. R. Maier S. F. (1995). A long-term increase in basal levels of corticosterone and a decrease in corticosteroid-binding globulin after acute stressor exposure. Endocrinology 136, 5336–5342 10.1210/en.136.12.5336 7588279

[B23] Fox K. R. (1999). The influence of physical activity on mental well-being. Public Health Nutr. 2, 411–418 10.1017/S1368980099000567 10610081

[B24] Giulian D. Baker T. J. Shih L. C. Lachman L. B. (1986). Interleukin 1 of the central nervous system is produced by ameboid microglia. J. Exp. Med. 164, 594–604 3487617 10.1084/jem.164.2.594 PMC2188228

[B25] Glass J. D. Dinardo L. A. Ethen J. C. (2000). Dorsal raphe nuclear stimulation of SCN serotonin release and circadian phase-resetting. Brain Res. 859, 224–232 10.1016/S0006-8993(00)01963-6 10719068

[B26] Gleeson M. Bishop N. C. Stensel D. J. Lindley M. R. Mastana S. S. Nimmo M. A. (2011). The anti-inflammatory effects of exercise:mechanisms and implications for the prevention and treatment of disease. Nat. Rev. Immunol. 11, 607–615 10.1038/nri3041 21818123

[B27] Grahn R. E. Will M. J. Hammack S. E. Maswood S. McQueen M. B. Watkins L. R. (1999). Activation of serotonin-immunoreactive cells in the dorsal raphe nucleus in rats exposed to an uncontrollable stressor. Brain Res. 826, 35–43 10.1016/S0006-8993(99)01208-1 10216194

[B28] Greenwood B. N. Fleshner M. (2011). Exercise, stress resistance, and central serotonergic systems. Exerc. Sport Sci. Rev. 39, 140–149 10.1097/JES.0b013e31821f7e45 21508844 PMC4303035

[B29] Greenwood B. N. Foley T. E. Day H. E. Burhans D. Brooks L. Campeau S. (2005). Wheel running alters serotonin (5-HT) transporter, 5-HT_1A_, 5-HT_1B_, and alpha 1b-adrenergic receptor mRNA in the rat raphe nuclei. Biol. Psychiatry 57, 559–568 10.1016/j.biopsych.2004.11.025 15737672

[B30] Greenwood B. N. Foley T. E. Day H. E. Campisi J. Hammack S. H. Campeau S. (2003). Freewheel running prevents learned helplessness/behavioral depression: role of dorsal raphe serotonergic neurons. J. Neurosci. 23, 2889–2898 12684476 10.1523/JNEUROSCI.23-07-02889.2003 PMC6742115

[B31] Greenwood B. N. Strong P. V. Loughridge A. B. Day H. E. Clark P. J. Mika A. (2012). 5-HT_2C_ receptors in the basolateral amygdala and dorsal striatum are a novel target for the anxiolytic and antidepressant effects of exercise. PLoS ONE 7:e46118 10.1371/journal.pone.0046118 23049953 PMC3458100

[B32] Grewal J. S. Mukhin Y. V. Garnovskaya M. N. Raymond J. R. Greene E. L. (1999). Serotonin 5-HT_2A_ receptor induces TGF-beta1 expression in mesangial cells via ERK:proliferative and fibrotic signals. Am. J. Physiol. 276, F922–F930 10362781 10.1152/ajprenal.1999.276.6.F922

[B33] Hale M. W. Shekhar A. Lowry C. A. (2012). Stress-related serotonergic systems: implications for symptomatology of anxiety and affective disorders. Cell. Mol. Neurobiol. 32, 695–708 10.1007/s10571-012-9827-1 22484834 PMC3378822

[B34] Hammack S. E. Richey K. J. Schmid M. J. LoPresti M. L. Watkins L. R. Maier S. F. (2002). The role of corticotropin-releasing hormone in the dorsal raphe nucleus in mediating the behavioral consequences of uncontrollable stress. J. Neurosci. 22, 1020–1026 11826130 10.1523/JNEUROSCI.22-03-01020.2002 PMC6758532

[B35] Hochstrasser T. Ullrich C. Sperner-Unterweger B. Humpel C. (2011). Inflammatory stimuli reduce survival of serotonergic neurons and induce neuronal expression of indoleamine 2,3-dioxygenase in rat dorsal raphe nucleus organotypic brain slices. Neuroscience 184, 128–138 10.1016/j.neuroscience.2011.03.070 21501664

[B36] Hökfelt T. Ljungdahl A. Steinbusch H. Verhofstad A. Nilsson G. Brodin E. (1978). Immunohistochemical evidence of substance P-like immunoreactivity in some 5-hydroxytryptamine-containing neurons in the rat central nervous system. Neuroscience 3, 517–538 10.1016/0306-4522(78)90017-9 358011

[B37] Holmes P. V. Yoo H. S. Dishman R. K. (2006). Voluntary exercise and clomipramine treatment elevate prepro-galanin mRNA levels in the locus coeruleus in rats. Neurosci. Lett. 408, 1–4 10.1016/j.neulet.2006.04.057 16996684

[B38] Kanehisa M. Goto S. (2000). KEGG:kyoto encyclopedia of genes and genomes. Nucleic Acids Res. 28, 27–30 10.1093/nar/28.1.27 10592173 PMC102409

[B39] Kelder T. van lersel M. P. Hanspers K. Kutmon M. Conklin B. R. Evelo C. T. (2012). Wikipathways:building research communities on biological pathways. Nucleic Acids Res. 40, D1301–D1307 10.1093/nar/gkr1074 22096230 PMC3245032

[B40] Kendler K. S. Karkowski L. M. Prescott C. A. (1999). Causal relationship between stressful life events and the onset of major depression. Am. J. Psychiatry 156, 837–841 10.1176/appi.pn.2013.2b29 10360120

[B41] Kim Y. K. Na K. S. Shin K. H. Jung H. Y. Choi S. H. Kim J. B. (2007). Cytokine imbalance in the pathophysiology of major depressive disorder. Prog. Neuropsychopharmacol. Biol. Psychiatry. 31, 1044–1053 10.1016/j.pnpbp.2007.03.004 17433516

[B42] Knol M. J. Twisk J. W. Beekman A. T. Heine R. J. Snoek F. J. Pouwer F. (2006). Depression as a risk factor for the onset of type 2 diabetes mellitus. A meta-analysis. Diabetologia 49, 837–845 10.1007/s00125-006-0159-x 16520921

[B43] Krishnan V. Nestler E. J. (2008). The molecular neurobiology of depression. Nature 455, 894–902 10.1038/nature07455 18923511 PMC2721780

[B44] Lindvall O. Björklund A. (1974). The organization of the ascending catecholamine neuron systems in the rat brain as revealed by the glyoxylic acid fluorescence method. Acta Physiol. Scand. Suppl. 412, 1–48 4531814

[B45] Lowry C. A. Rodda J. E. Lightman S. L. Ingram C. D. (2000). Corticotropin-releasing factor increases *in vitro* firing rates of serotonergic neurons in the rat dorsal raphe nucleus: evidence for activation of a topographically organized mesolimbocortical serotonergic system. J. Neurosci. 20, 7728–7736 11027235 10.1523/JNEUROSCI.20-20-07728.2000 PMC6772886

[B46] Maes M. (2008). The cytokine hypothesis of depression: inflammation, oxidative & nitrosative stress (IO&NS) and leaky gut as new targets for adjunctive treatments in depression. Neuro Endocrinol. Lett. 29, 287–291 18580840

[B47] Maier S. F. Grahn R. E. Watkins L. R. (1995). 8-OH-DPAT microinjected in the region of the dorsal raphe nucleus blocks and reverses the enhancement of fear conditioning and the interference with escape produced by exposure to inescapable shock. Behav. Neurosci. 109, 404–413 7662151 10.1037//0735-7044.109.3.404

[B48] Maier S. F. Watkins L. R. (1995). Intracerebroventricular interleukin-1 receptor antagonist blocks the enhancement of fear conditioning and interference with escape produced by inescapable shock. Brain Res. 695, 279–282 10.1016/0006-8993(95)00930-O 8556346

[B49] Maier S. F. Watkins L. R. (1998). Stressor controllability, anxiety, and serotonin. Cogn. Therapy Res. 22, 595–613

[B50] Maier S. F. Watkins L. R. (2005). Stressor controllability and learned helplessness: the roles of the dorsal raphe nucleus, serotonin, and corticotropin-releasing factor. Neurosci. Biobehav. Rev. 29, 829–841 10.1016/j.neubiorev.2005.03.021 15893820

[B51] Marinelli S. Schnell S. A. Hack S. P. Christie M. J. Wessendorf M. W. Vaughan C. W. (2004). Serotonergic and nonserotonergic dorsal raphe neurons are pharmacologically and electrophysiologically heterogeneous. J. Neurophysiol. 92, 3532–3537 10.1152/jn.00437.2004 15254076

[B52] Mathers C. D. Loncar D. (2006). Projections of global mortality and burden of disease from 2002 to 2030. PLoS Med. 3:e442 10.1371/journal.pmed.0030442 17132052 PMC1664601

[B53] Merlio J. P. Ernfors P. Jaber M. Persson H. (1992). Molecular cloning of rat trkC and distribution of cells expressing messenger RNAs for members of the trk family in the rat central nervous system. Neuroscience 51, 513–532 10.1016/0306-4522(92)90292-A 1488112

[B54] Miller J. A. Oldham M. C. Geschwind D. H. (2008). A systems level analysis of transcriptional changes in Alsheimer's disease and normal aging. J. Neurosci. 28, 1410–1420 10.1523/JNEUROSCI.4098-07.2008 18256261 PMC2902235

[B55] Moraska A. Campisi J. Nguyen K. T. Maier S. F. Watkins L. R. Fleshner M. (2002). Elevated IL-1beta contributes to antibody suppression produced by stress. J. Appl. Physiol. 93, 207–215 10.1152/japplphysiol.01151.2001 12070207

[B56] Nair A. Bonneau R. H. (2006). Stress-induced elevation of glucocorticoids increases microglia proliferation through NMDA receptor activation. J. Neuroimmunol. 171, 72–85 10.1016/j.jneuroim.2005.09.012 16278020

[B57] Neeper S. A. Gómez-Pinilla F. Choi J. Cotman C. (1995). Exercise and brain neurotrophins. Nature 373:109 10.1038/373109a0 7816089

[B58] Nguyen K. T. Deak T. Owens S. M. Kohno T. Fleshner M. Watkins L. R. (1998). Exposure to acute stress induces brain interleukin-1beta protein in the rat. J. Neurosci. 18, 2239–2246 9482808 10.1523/JNEUROSCI.18-06-02239.1998 PMC6792918

[B59] Okamoto M. Hojo Y. Inoue K. Matsui T. Kawato S. McEwen B. S. (2012). Mild exercise increases dihydrotestosterone in hippocampus providing evidence for androgenic mediation of neurogenesis. Proc. Natl. Acad. Sci. U.S.A. 109, 13100–13105 10.1073/pnas.1210023109 22807478 PMC3420174

[B60] Rapaport M. H. Clary C. Fayyad R. Endicott J. (2005). Quality-of-life impairment in depressive and anxiety disorders. Am. J. Psychiatry 162, 1171–1178 10.1176/appi.ajp.162.6.1171 15930066

[B61] Righi M. Mori L. De Libero G. Sironi M. Biondi A. Mantovani A. (1989). Monokine production by microglial cell clones. Eur. J. Immunol. 19, 1443–1448 10.1002/eji.1830190815 2789141

[B62] Rozeske R. R. Evans A. K. Frank M. G. Watkins L. R. Lowry C. A. Maier S. F. (2011). Uncontrollable, but not controllable, stress desensitizes 5-HT_1A_ receptors in the dorsal raphe nucleus. J. Neurosci. 31, 14107–14115 21976495 10.1523/JNEUROSCI.3095-11.2011 PMC3207271

[B63] Rusak B. Zucker I. (1979). Neural regulation of circadian rhythms. Physiol. Rev. 59, 449–526 379886 10.1152/physrev.1979.59.3.449

[B64] Safran M. Dalah I. Alexander J. Rosen N. Iny Stein T. Shmoish M. (2010). GeneCards Version 3: the human gene integrator. Database(Oxford). Available online at: www.genecards.org Accessed March 2012. 10.1093/database/baq020 20689021 PMC2938269

[B65] Sawada M. Kondo N. Suzumura A. Marunouchi T. (1989). Production of tumor necrosis factoralpha by microglia and astrocytes in culture. Brain Res. 491, 394–397 10.1016/0006-8993(89)90078-4 2765895

[B66] Sciolino N. R. Dishman R. K. Holmes P. V. (2012). Voluntary exercise offers anxiolytic potential and amplifies galanin gene expression in the locus coeruleus of the rat. Behav. Brain Res. 233, 191–200 10.1016/j.bbr.2012.05.001 22580167 PMC3409590

[B67] Sharp T. Cowen P. J. (2011). 5-HT and depression: is the glass half-full? Curr. Opin. Pharmacol. 11, 45–51 10.1016/j.coph.2011.02.003 21377932

[B68] Sherman A. D. Sacquitne J. L. Petty F. (1982). Specificity of the learned helplessness model of depression. Pharmacol. Biochem. Behav. 16, 449–54 7200610 10.1016/0091-3057(82)90451-8

[B69] Soh D. Dong D. Guo Y. Wong L. (2010). Consistency, comprehensiveness, and compatibility of pathway databases. BMC Bioinformatics 11:449 10.1186/1471-2105-11-449 20819233 PMC2944280

[B70] Song C. Earley B. Leonard B. E. (1996). The effects of central administration of neuropeptide Y on behavior, neurotransmitter, and immune functions in the olfactory bulbectomized rat model of depression. Brain Behav. Immun. 10, 1–16 10.1006/brbi.1996.0001 8735565

[B71] Speaker K. J. Greenwood B. N. Fleshner M. (2011). Regular, moderate exercise attenuates the stress induced increase in plasma IL-1β but not TNF-α, IL-6, IL-10 or corticosterone. S. 10, in International Society of Exercise Immunology Biannual Conference (Oxford).

[B72] Spiritzer M. D. Galea L. A. (2007). Testosterone and dihydrotestosterone, but not estradiol, enhance survival of new hippocampal neurons in adult male rats. Dev. Neurobiol. 67, 1321–1333 10.1002/dneu.20457 17638384

[B73] Sprouse J. S. Aghajanian G. K. (1987). Electrophysiological responses of serotoninergic dorsal raphe neurons to 5-HT_1A_ and 5-HT_1B_ agonists. Synapse 1, 3–9 10.1002/syn.890010103 3505364

[B74] Stratford T. R. Wirtshafter D. (1990). Ascending dopaminergic projections from the dorsal raphe nucleus in the rat. Brain Res. 511, 173–176 10.1016/0006-8993(90)90239-8 1970510

[B75] Sugama S. Fujita M. Hashimoto M. Conti B. (2007). Stress induced morphological microglial activation in the rodent brain: involvement of interleukin-18. Neuroscience 146, 1388–1399 10.1016/j.neuroscience.2007.02.043 17433555

[B76] Swanson L. W. Sawchenko P. E. Rivier J. Vale W. W. (1983). Organization of ovine corticotropin-releasing factor immunoreactive cells and fibers in the rat brain: an immunohistochemical study. Neuroendocrinology 36, 165–186 6601247 10.1159/000123454

[B77] Tao R. Auerbach S. B. (2000). Regulation of serotonin release by GABA and excitatory amino acids. J. Psychopharmacol. 14, 100–113 10.1177/026988110001400201 10890306

[B78] Thompson R. S. Christianson J. P. Maslanik T. M. Maier S. F. Greenwood B. N. Fleshner M. (2013). Effects of stressor controllability on diurnal physiological rhythms. Physiol. Behav. 112–113, 32–39 10.1016/j.physbeh.2013.02.009 23454291 PMC3637963

[B79] Tong L. Shen H. Perreau V. M. Balazs R. Cotman C. W. (2001). Effects of exercise on geneexpression profile in the rat hippocampus. Neurobiol. Dis. 8, 1046–1056 10.1006/nbdi.2001.0427 11741400

[B80] Valentino R. J. Bey V. Pernar L. Commons K. G. (2003). Substance P Acts through local circuits within the rat dorsal raphe nucleus to alter serotonergic neuronal activity. J. Neurosci. 23, 7155–7159 12904475 10.1523/JNEUROSCI.23-18-07155.2003 PMC6740675

[B81] van Praag H. M. (2005). Can stress cause depression? World J. Biol. Psychiatry. 6 Suppl 2, 5–22 10.1080/15622970510030018 16166019

[B80a] Wulsin L. R. Singal B. M. (2003). Do depressive symptoms increase the risk for the onset of coronary disease? A systematic quantitative review. Psychosom. Med. 65, 201–210 10.1097/01.PSY.0000058371.50240.E3 12651987

[B81b] Xu Z. Q. Hökfelt T. (1997). Expression of galanin and nitric oxide synthase in subpopulations of serotonin neurons of the rat dorsal raphe nucleus. J. Chem. Neuroanat. 13, 169–187 10.1016/S0891-0618(97)00043-4 9315967

[B81c] Xu Z. Q. Zhang X. Pieribone V. A. Grillner S. Hökfelt T. (1998). Galanin-5-hydroxytryptamine interactions: electrophysiological, immunohistochemical and *in situ* hybridization studies on rat dorsal raphe neurons with a note on galanin R1 and R2 receptors. Neuroscience 87, 79–94 10.1016/S0306-4522(98)00151-1 9722143

